# Microenvironmentally-driven Plasticity of CD44 isoform expression determines Engraftment and Stem-like Phenotype in CRC cell lines

**DOI:** 10.7150/thno.39893

**Published:** 2020-06-18

**Authors:** Thiemo F. Dinger, Oleg Chen, Claudia Dittfeld, Lydia Hetze, Melanie Hüther, Marit Wondrak, Steffen Löck, Wolfgang Eicheler, Georg Breier, Leoni A. Kunz-Schughart

**Affiliations:** 1 OncoRay - National Center for Radiation Research in Oncology, Faculty of Medicine and University Hospital Carl Gustav Carus, TU Dresden, and Helmholtz-Zentrum Dresden-Rossendorf, Germany.; 2 Department of Neurosurgery, University Hospital Essen, Faculty of Medicine, University of Duisburg-Essen, Germany.; 3 Department of Cell Signaling, Institute of Cell Biology, National Academy of Sciences of Ukraine, Lviv, Ukraine.; 4 Department of Cardiac Surgery, Herzzentrum Dresden, Faculty of Medicine Carl Gustav Carus, TU Dresden, Germany.; 5 German Cancer Consortium (DKTK), Partner Site Dresden, and German Cancer Research Center (DKFZ), Heidelberg, Germany.; 6 Department of Radiation Oncology, University Hospital Carl Gustav Carus, Dresden, Germany.; 7 Division of Medical Biology, Department of Psychiatry, Faculty of Medicine and University Hospital Carl Gustav Carus, TU Dresden, Germany.; 8 National Center for Tumor Diseases (NCT), partner site Dresden, Germany.

**Keywords:** microenvironment, CD44/CD44v8-10, colorectal cancer, limiting dilution engraftment, fibroblasts

## Abstract

Theranostic biomarkers for putative cancer stem-like cells (CSC) in colorectal cancer (CRC) are of particular interest in translational research to develop patient-individualized treatment strategies. Surface proteins still under debate are CD44 and CD133. The structural and functional diversity of these antigens, as well as their plasticity, has only just begun to be understood. Our study aimed to gain novel insight into the plasticity of CD133/CD44, thereby proving the hypothesis of marker-associated tumorigenic and non-tumorigenic phenotypes to be environmentally driven.

**Methods:** CD133/CD44 profiles of 20 CRC cell lines were monitored; three models with distinct surface patterns *in vitro* were systematically examined. CD133/CD44 subpopulations were isolated by FACS and analyzed upon *in vitro* growth and/or in limiting dilution engraftment studies. The experimental setup included biomarker analyses on the protein (flow cytometry, Western blotting, immunofluorescence) and mRNA levels (RT-/qPCR) as well as CD44 gene sequencing.

**Results:** In general, we found that (i) the *in vitro* CD133/CD44 pattern never determined engraftment and (ii) the CD133/CD44 population distributions harmonized under *in vivo* conditions. The LS1034 cell line appeared as a unique model due to its *de novo in vivo* presentation of CD44. *CD44v8-10* was identified as main transcript, which was stronger expressed in primary human CRC than in normal colon tissues. Biomarker pattern of LS1034 cells* in vivo* reflected secondary engraftment: the tumorigenic potential was highest in CD133^+^/CD44^+^, intermediate in CD133^+^/CD44^-^ and entirely lost in CD133^-^/CD44^-^ subfractions. Both CD44^+^ and CD44^-^ LS1034 cells gave rise to tumorigenic and non-tumorigenic progeny and were convertible - but only as long as they expressed CD133* in vivo*. The highly tumorigenic CD133^+^/CD44(v8-10)^+^ LS1034 cells were localized in well-oxygenated perivascular but not hypoxic regions. From a multitude of putative modulators, only the direct interaction with stromal fibroblasts triggered an essential, *in vivo*-like enhancement of CD44v8-10 presentation *in vitro*.

**Conclusion:** Environmental conditions modulate CD133/CD44 phenotypes and tumorigenic potential of CRC subpopulations. The identification of fibroblasts as drivers of cancer-specific CD44 expression profile and plasticity sheds light on the limitation of per se dynamic surface antigens as biomarkers. It can also explain the location of putative CD133/CD44-positive CRC CSC in the perivascular niche, which is likely to comprise cancer-associated fibroblasts. The LS1034 *in vitro/in vivo* model is a valuable tool to unravel the mechanism of stromal-induced CD44v8-10 expression and identify further therapeutically relevant, mutual interrelations between microenvironment and tumorigenic phenotype.

## Introduction

Colorectal cancers (CRC) are proposed to not exclusively develop according to the stochastic clonal evolution model analog to the Darwinian principle, but appear to some extent be hierarchically organized with undifferentiated, highly tumorigenic, self-renewing cancer stem-like cell (CSC) population(s) and more differentiated, non-tumorigenic progeny 1-3. However, accumulating evidence suggests that stemness and non-stemness phenotypes are spatiotemporally plastic and can be driven by environmental constraints 4-7.

Various biomarkers such as CD44, CD133, CD166, ALDH, or Lgr5 have shown the potential to enrich CSC from CRC 8-15. Nonetheless, the regulation, structural diversity, and multifunctionality of the majority of these putative CSC markers need to be better understood to prove their theranostic merit. Intestinal stem cells in human and murine tissue are known to express both standard (CD44s) and variant CD44 (CD44v) isoforms. The latter were described to promote adenoma formation in mouse models of familial adenomatous polyposis 16. Human CRC cells, including established cell lines, express different CD44 variants that may or may not correlate with metastasis, high recurrence or poor survival in CRC patients 17-20. Determining the role of CD44 in CRC stemness and tumorigenicity thus remains challenging due to the potential co-expression of splice variants and functionally distinct isoforms 9 as well as the putative environmentally and/or epigenetically driven marker plasticity.

An additional dilemma for theranostically-relevant CSC research is the postulated need for primary patient-derived tumor material and cells, respectively. Although established CRC cell lines were recently shown to express proteome profiles representative of primary tumors and to predict drug sensitivity 21, they are often considered to reflect neither biomarker-correlated tumorigenic behavior nor phenotypic transition between CSC and non-CSC 22. Despite this highly-charged debate, cell lines are still basis for genetically engineered cell clones and of great value for large therapy screening initiatives.

To better elucidate the outlined limitations and challenges, we pre-screened 20 CRC cell lines for their CD44/CD133 surface profiles under identical, standardized conditions and choose three models with strikingly distinct CD44/CD133 surface pattern for extended, systematic *in vitro/in vivo* examination. Amongst others, our experimental design included limiting dilution engraftment studies of cell line subpopulations after fluorescence-activated cell sorting (FACS), biomarker analyses on protein (flow cytometry, Western blotting, immunofluorescence) and mRNA levels (RT-PCR) *in vitro* and *in vivo* as well as CD44 gene sequencing. We gained insight into the plasticity of CD133/CD44 expression, in particular in the unique LS1034 cell line model, thereby addressing novel aspects underlining the relevance of the stromal tumor microenvironment for engraftment and phenotypic interconversion.

## Methods

### Cell lines and routine culture conditions

Numerous CRC cell lines were examined, in particular LS1034, SW480, and SW620, all obtained from the ATCC (American Type of Culture Collection, USA). Authentication of the entire CRC cell line panel (e.g., Figure S1A) was performed with multiplex PCR kits, i.e., Mentype® NonaplexQS Twin (Biotype) and the PowerPlex® 16 System (Promega), at the Institute of Legal Medicine (TU Dresden, Germany) as detailed earlier 23. Cultures were tested free of mycoplasmas using a PCR Mycoplasma Kit (Applichem) and were routinely grown from the validated frozen stocks for 2 to a maximum of 20 passages (<120 cumulative population doublings) for experimental setup. All cell lines were cultured at 37 °C in a humidified 8% CO_2_ atmosphere using standard DMEM with L-glutamine, D-glucose (1 g/L) and 25 mM HEPES supplemented with 10% heat-inactivated FCS and 1% penicillin/streptomycin (10,000 U/mL / 10 mg/mL). Single-cell suspensions for *in vitro* and *in vivo* application were obtained from exponentially growing cultures by mild enzymatic and mechanic means using a 0.05% trypsin / 0.02% EDTA solution in phosphate buffered saline (PBS). For LS1034 cell detachment, the enzyme cocktail was further supplemented with collagenase III in a 1:500 dilution of the stock solution. All media, supplements, and solutions for cell culturing were from PAN Biotech if not stated otherwise. A CASY® TTC device (Roche Innovatis) was utilized for cell counting, cell volume analysis, and culture quality assessment.

### Modification of 2-D and 3-D culture environment

LS1034 cells were monitored *in vitro* for CD44 surface expression under various physiological and pathophysiological conditions. Cells were grown in exponential, non-confluent, confluent, and post-confluent 2-D cultures as well as in small clusters or spheres and spheroids of different sizes by modifying culture vessel and surface coating, cell densities, and culture medium with supplements. In addition to standard DMEM (see above) with and without 10% FCS, we applied (i) neurobasal medium conditioned with 2% B27 supplement, 0.5 mM Glutamax, 1 mM sodium pyruvate (all from Life Technologies) plus 10 ng/mL EGF (R&D Systems) and 10 ng/mL FGF-2 (PreproTech) as stem cell medium 1 (SC1), and (ii) MEBM (mammary epithelial cell basal medium; Lonza) containing 1% Penicillin/Streptomycin, 2% B27 supplement, 20 ng/mL EGF, 20 ng/mL FGF, and 4 µg/mL insulin (Sigma-Aldrich) as stem cell medium 2 (SC2).

Cells were cultured in T25 culture flasks, 10 cm dishes, 6-well plates, and 96-well plates. Commercial 6-well plates without and with poly-D-lysin, fibronectin, laminin, collagen type I, or collagen type IV coating (Corning® BioCoat™) were used. Other 6-well plates were manually pre-coated with 0.1 -1.0 mg/mL hyaluronic acid solution (ACROS Organics™) according to Corradetti et al. 24 for 2-D culturing or with a hyaluronic acid scaffold (HyStem® Cell Culture Scaffold Kit, Sigma-Aldrich) for 3-D spheres. 96-well plates (Corning) were coated with 1.5% agarose in serum-free medium for liquid overlay spheroid culturing as previously described 25. Spheroids of different sizes (400 µm to >800 µm) at day 4 in culture were prepared by seeding increasing cell numbers per well.

Cells grown in DMEM were routinely kept at 8% CO_2_ in a humidified air condition, which contains about 18-19% O_2_ according to Place et al. 26; some monolayer cultures were exposed to 4% O_2_ in a BioSpa 8 system (BioTek Instruments) to reflect tissue normoxia in the colon. The normal pH in the culture medium was 7.2-7.4; pH modifications included mild (pH 6.9±0.1) and harsh (pH 6.4±0.1) acidosis achieved in standard medium by adding either hydrochloric acid or 10-20 mM lactic acid (Sigma-Aldrich). The latter mimics the pathophysiological accumulation of lactate observed in colorectal and other solid cancers 27,28. HEPES and MES-based buffer systems were adapted from Park et al. 29 and Sørensen et al. 30 to keep the low pH stable over a period of several days, thereby avoiding unphysiological modifications in osmolarity. Milieu conditions were modified by medium exchange after cell attachment (6-8 h after seeding); cells for 2-D culturing under harsh conditions or in serum-free medium were seeded at higher concentrations to account for considerable cell death and to obtain (0.5-1)×10^6^ cells/dish on the day of measurement.

LS1034 cells were also exposed to IL-6 by seeding 1.2×10^5^ tumor cells per well in 6-well plates using standard DMEM with FCS supplemented with 10, 50, or 100 ng/mL IL-6. Finally, co-culture experiments were carried out by pre-seeding 7×10^4^ or 1.2×10^5^ normal skin (VF2) and colon cancer-derived fibroblasts (CF) or up to 4×10^5^ human umbilical vein endothelial cells (HUVEC) per well into 6-well plates and adding 1.2×10^5^ LS1034 cells 24 h later. In some cases, HUVEC were mixed with fibroblasts at a 1:15 ratio similar to an established angiogenesis assay protocol 31. Preparation and culturing of low-passage fibroblasts and HUVEC was performed as described earlier 31,32. Co-culturing was carried out in either standard DMEM with 10% FCS or in supplemented EGM-2 (endothelial cell growth medium 2) containing 2% FCS as well as recombinant human EGF, bFGF, and VEGF, IGF, ascorbic acid, heparin hydrocortisone (EGM-2 and supplement from PromoCell). 6-well plates with inserts (0.4 µm pore size, Costar, Greiner bio-one) were applied for non-contact co-culturing of LS1034 cells with VF2 fibroblasts. Here, up to 4×10^5^ fibroblasts were seeded into the insert and pre-cultured for 24 h before adding 1.2×10^5^ cancer cells to the bottom well.

Notably, a 5% CO_2_ in air atmosphere was used for all specific media (SC1, SC2, EGM-2) as recommended by the manufacturers, in contrast to the 8% CO_2_ incubator setup for DMEM. If not stated otherwise, cells were detached and analyzed for CD44 surface expression by flow cytometry (see below) after 4 days of exposure to the respective conditions. Cell suspensions from co-cultures were co-stained for CD326 to discriminate stromal cell fractions from LS1034 cells.

### Flow cytometric (FC) analyses and fluorescence-activated cell sorting (FACS)

Single-cell suspensions from cultures or xenografts were labeled with APC-conjugated anti-panCD44 and PE-conjugated anti-CD133-1 (AC133) antibodies followed by two FASER steps for CD133 detection as highlighted 33. Xenograft samples were additionally labeled with either anti-human CD326 or anti-HLA antibodies. Aliquots exposed to isotype-control antibodies were always analyzed in parallel. Antibodies and preparative details are listed in Table S1. Propidium iodide (PI; 2 µg/mL) was added before measurement for dead-cell discrimination. A minimum of 2×10^4^ events and >1.5×10^4^ membrane-intact human cells, respectively, were analyzed per sample. FC stain indices (SI) were calculated to allow the comparison of fluorescence intensities reflecting CD133 or CD44 surface levels in different cells and subpopulations. According to FC best practice, SI values were calculated as described earlier 34,35 via the following function (FL - fluorescence signal; SD - standard deviation):

SI = (median FL_stained cell (sub)population_ - median FL_isotype_) / 2×SD of FL_isotype_

For intracellular FC analyses, single-cell suspensions were fixed in a 4% paraformaldehyde solution (Honeywell Fluka) for 1-12 h at 4 °C, then washed and permeabilized in 0.05 g/L saponin (Honeywell Fluka) 0.19 g/L HEPES buffer. After another washing step with PBS, cells were stained with antibodies and measured according to the standard protocol; PI was not added in this case. A FACSCanto™ (BD Biosciences) was used for most FC analyses.

For FACS, single-cell suspensions stained as detailed were re-suspended at a concentration of (0.5-1)×10^7^ cells/mL in PBS with 2 mM EDTA. PI-negative cells with defined surface expression pattern were sorted using a FACSAria™ II (BD Biosciences). Subpopulations were routinely re-analyzed immediately after sorting to reveal a purity of ≥96% before further use. Note: The real contaminating cell fraction after sorting was lower than indicated by this purity value since the fluorescence signal of the positive fractions in general slightly shifted to the left when being exposed to the laser a second time due to partial bleaching; this resulted in a higher signal overlap of isotype control and positive populations, thereby reducing the sensitivity for discrimination of subpopulations. Recovery of samples sorted from *in vitro* cultures were re-assessed with the Casy1 cell analyzer, whereas samples originated from dissociated xenografts were counted manually with a Neubauer hemocytometer (Brand) using trypan blue (0.5% in physiological saline solution; Biochrom) in a 1:2 to 1:5 dilution to discriminate membrane-defect cells prior to re-culturing or *in vivo* implantation of defined viable cell numbers.

The FACS setup to isolate CD44-positive and -negative LS1034 fractions from CD44 highly-positive fibroblasts in co-culture suspensions included labeling with a PE-conjugated panCD44 Ab and a FITC-conjugated CD326-specific Ab combined with SYTOX staining (working concentration: 1 µM, SYTOX™ Blue Dead Cell Stain, Thermo Fisher Scientific) for dead cell exclusion, and the utilization of a FACSAria™ Fusion (BD Biosciences) for sorting. Samples were then further processed for RNA extraction and RT-PCR analyses. Selected cell suspensions from co-cultures were also co-stained using a specific primary Ab directed against CD44v9 combined with an Alexa Fluor™ 405-conjugated secondary Ab prior to labeling for total CD44 (PE) and CD326 (FITC). In this case, PI was applied for discriminating membrane-defect cells as described before, and FC measurements were performed on a FACSCelesta™ (BD Biosciences).

### *In vitro* re-culturing of sorted subpopulations

A total of 6.5×10^4^ (4 days) or 1×10^4^ (9 & 18 days) FACSorted CD133^+^/CD44^+^, CD133^+^/CD44^-^, CD133^-^/CD44^+^, CD133^-^/CD44^-^, or original (run through sorter) SW620 cells were seeded into monolayer culture vessels at a density of 1×10^3^ cells/cm². On days 4, 9, and 18 after seeding, single-cell suspensions were obtained by enzymatic dissociation and cell numbers were determined in biological triplicates. Simultaneously, cells were analyzed for their CD133/CD44 cell surface expression via FC (see above).

### Limiting dilution xenograft formation assay (primary and secondary engraftment)

The *in vivo* experiments were performed using female NMRI (nu/nu) mice. The animal facility (Experimental Center, Medical Faculty, TU Dresden) and all animal studies were approved according to institutional guidelines and German animal welfare regulations.

Two to five days before tumor cell injection, mice underwent whole-body irradiation with 4 Gy for immune suppression using a radiation-shielded cabinet MaxiShot 200 with a XYLON.TU 320-D03 x-ray tube (both Xylon International; 200 kV X-rays, 1.2-1.3 Gy/min dose-rate). SW480, SW620 or LS1034 single-cell suspensions derived from unsorted or sorted monolayer and xenograft samples, respectively, were prepared with defined concentrations of 10 - 10^5^ cells per 70 µl of a 50% Matrigel (BD Biosciences) in PBS solution and (re-)injected subcutaneously into the hind limbs of the animals. Xenograft formation and size were routinely monitored by examining the mice two to three times a week for a period of 120 days after injection as described earlier 33. Primary LS1034 cell line xenografts for secondary engraftment studies were harvested, dissociated, stained, sorted, re-analyzed, and re-injected into next generation mice using similar limiting dilution approaches and identical monitoring periods. To avoid interpretation errors due to preparative artifacts, our animal experimental design included 2-4 entirely independent cell preparations for each CD133/CD44-defined subfraction/subpopulation used for injections with limiting dilution. Furthermore, correct assessment of tumor take rates (TTR) with adequate post-injection monitoring times required a standardized experimental setup with only one injection site per mouse.

### Xenograft processing

Xenografts with a mean diameter of 1.1 - 1.3 cm were used for extended cell and tissue analyses as well as secondary engraftment. Mice selected for fluorescence microscopic tumor tissue monitoring received an intraperitoneal pimonidazole injection (Natural Pharmacia International, Belmont, MA, USA; 0.1 mg/g body weight, dissolved at 10 mg/mL in 0.9% NaCl) 45 min before sacrifice and xenograft excision. Excised tissue material was then immediately shock-frozen in liquid nitrogen for cryosectioning and immunofluorescence stainings. Xenografts for FC analyses were dissected, non-tumor tissue was removed, and tumor material was minced with scalpels. Depending on the cell line model, xenografts were dissociated either by exposure to Collagenase NB 4G (0.5 U/mL; Serva), hyaluronidase (0.05 mg/mL) and DNAse (0.01 mg/mL; both from Sigma-Aldrich) at 37 °C overnight (SW480, SW620) or using Collagenase NB 4G (0.5 U/mL) plus 0.25% trypsin / 0.1% EDTA in PBS for 70 min at 37 °C (LS1034). The resulting cell suspensions were filtered through a 70 µm mesh, and viable cell numbers were determined in the Neubauer hemocytometer upon trypan blue staining. Aliquots were stained for FC and FACS as described above. Selected freshly excised xenografts were used in total or as pieces (1/2) for protein extraction to perform Western blot analyses (sample ID affix: -P). RNA for RT-PCR was prepared from different xenografts samples either directly after excision or following shock-freezing and storage of the tissues in liquid N_2_ (sample ID affix: -R).

### Histomorphology and immunostaining of xenografts

For histomorphological observation, cryosections (10 µm) of shock-frozen SW620, SW480, and LS1034 xenografts were stained with hematoxylin-eosin (H&E) and documented using an AxioImager M1 microscope (Carl Zeiss MicroImaging). For immunofluorescence stainings, frozen sections were fixed with acetone (10 min, 4 °C; Merck Millipore) and stored for 48 h at 4 °C. To visualize the distribution of CD44 positive cells relative to vessels and hypoxic areas, sections were exposed to a 1% BSA (Biomol) in PBS blocking solution for 1 h at room temperature and then stained for CD44, CD31, and pimonidazole. Antibodies with concentrations and incubation times for immunostaining are given in Table S1. Adequate isotype controls were routinely applied on parallel sections. After nuclear counterstaining with DAPI, slides were mounted (Dako Fluorescent Mounting Medium; Dako) and stored for 12 h at 4 °C in the dark before visualization on a Zeiss - AxioImager M1 microscope. Images were taken as mosaics with a 40× objective and processed with the ZEN blue 2012 software (both Carl Zeiss MicroImaging) to merge total views of median xenograft sections.

### Western blotting (WB)

Whole cell protein was extracted from cell cultures and xenografts using a RIPA buffer containing 0.1 mM PMSF, protease inhibitor cocktails 1+2 (1:100; P2850 + P5726; Sigma-Aldrich), complete mini protease inhibitor cocktail (1:10; Roche), 1 mM Na_3_VO_4_, and 1 mM NaF. Protein content was determined with the BCA protein assay kit (Pierce) as described by the manufacturer, and aliquots were stored at -80 °C. Proteins were separated by SDS-PAGE (8%) and transferred onto nitrocellulose membranes (Whatman); 25 - 50 µg of protein from *in vitro* cultures and 50 µg of protein from *in vivo* samples were loaded. Two different antibodies were used to visualize the expression of CD44 (see Table S1 for details); β-actin or α-tubulin served as loading controls. An anti-human MHC class I + HLA B antibody was applied as human-specific loading control for xenograft-derived samples with putative murine contamination.

### PCR analyses (Reverse transcription (RT) and quantitative (q)-PCR analyses)

Total cellular RNA was isolated from cell cultures and xenograft materials using the RNeasy Mini kit (QIAGEN) according to the manufacturer's protocol. RNA concentrations and quality were verified with a Nanodrop 1000 spectrophotometer (Thermo Fisher Scientific). The Verso cDNA Synthesis Kit (Thermo Fisher Scientific) and 1 µg of each total RNA were applied for cDNA synthesis; the PCR was carried out in an MJ Research PTC-200 Thermal Cycler (Bio-Rad) using the GoTaq Flexi DNA Polymerase Kit (Promega) with human specific primers for *ACTB, B2M, CD44* total, *CD44* isoforms, and *CD44 v9* exon. Conditions for the PCR were as follows: initial denaturation 95 °C for 7 min, denaturation in cycle 95 °C for 30 s, annealing 55 °C for 30 s, synthesis 72 °C for 1 min, and the final extension step was 72 °C for 7 min. PCR products and GeneRuler 100 bp Plus DNA Ladder (Thermo Fisher Scientific) were separated by 2.0 % agarose gel electrophoresis, visualized by RedSafe (iNtRON Biotechnology) dye staining, and documented using the GeneGenius Gel Imaging System (Syngene, UK). Human-specific *ACTB* and *B2M* gene primers were applied as reference control. Selected PCR products were identified by re-extraction from agarose gels and direct sequencing (sequence analysis by Eurofins genomics).

For quantitative (q)-PCR analysis, cDNA was diluted (1:10), and the one step PCR reaction was performed using the GoTaq qPCR Master Mix (Promega) according to the manufacturer's protocol. Data were collected and analyzed using the Applied Biosystems StepOnePlus Real-Time PCR System with v.2.2.2 StepOne Software (Life Technologies, Applied Biosystems). The relative gene expression of control versus treated cells was assessed by the comparative threshold cycle (ΔΔC_t_) method using *ACTB* as reference control; values ≥2 fold were considered as differentially regulated. All primer pairs with product sizes and number of cycles are listed in Table S2.

### Statistical analyses

Logistic regression, including Bonferroni-Holm correction, was employed to predict tumor take rates (TTR) from the logarithm of the injected cell numbers for each cell line and subpopulation, respectively. Differences in D_50_ (cell number leading to a 50% engraftment) were tested for statistical significance by a bootstrapping approach in Stata 11.2. 10,000 bootstraps were generated, and the D_50_ ratios of two groups of interest were calculated for each run. A statistically significant difference occurred if the corresponding 95% confidence interval did not include the value 1.

Further statistical analysis was performed using the survival package in R (release 3.4.1, https://www.r-project.org). Tumor control (TC) as function of time post-injection was estimated by the Kaplan-Meier method with tumor growth being the only defined endpoint. TC reflects tumor-free survival, with a few mice been censored due to non-tumor related death before the end of the observation period. These mice were excluded in the TTR documentation and evaluations. TC in different groups was compared by the log-rank test. Significance levels for all analyses are documented as p<0.05 (*), p<0.01 (**), and p<0.001 (***).

Xenografts after injections of low cell numbers develop with considerable delay and are palpable after highly variable lag phases. Data normalization was thus required to account for the intra-group variations as a prerequisite for comparing volume growth kinetics. For this normalization, the day when each individual xenograft exceeded a lower threshold of 5 mm in mean diameter (>66 µm³ in volume) was set to 1. Volume data for each xenograft group of interest, i.e., derived from the same subfraction and injected cell number, were averaged for overlapping monitoring intervals and are documented with standard deviations for both volume (y axis) and time (x axis). Calculations were generally based on all developed xenografts per group, except for the 500 cell injections of CD133^+^ and CD133^-^ LS1034 *in vitro* cells where 1/5 growing tumors in each group had to be excluded due to insufficient long-term monitoring time points.

Bioinformatic analysis of the TCGA (The Cancer Genome Atlas) and GTEx (The Genotype-Tissue Expression) colon datasets was conducted on processed RNA-seq array, gene alteration data, and paired clinical feature data through the UCSC Xena platform (https://xenabrowser.net). Statistical values were generated automatically in the UCSC Xena browser.

## Results

### CD44/CD133 surface pattern *in vitro* does not determine CRC cell line engraftment

**Figure 1:**
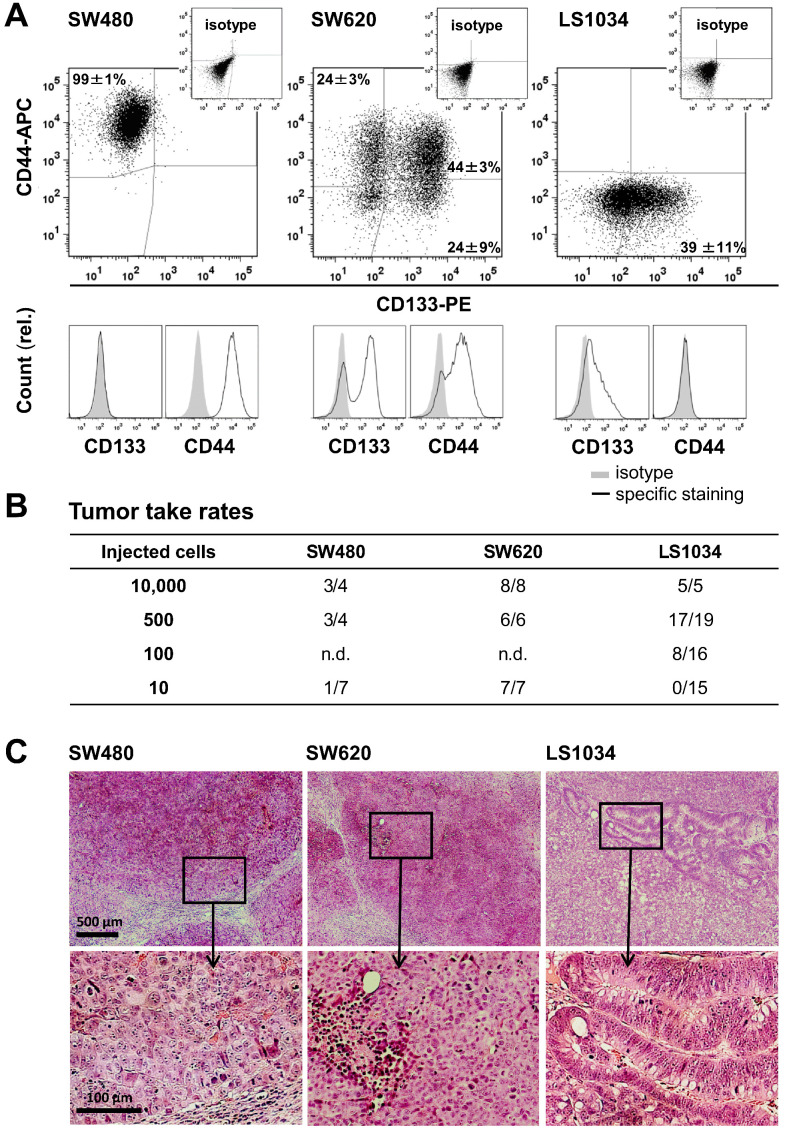
** CRC cell lines show heterogeneous CD133/CD44 surface expression pattern not reflecting engraftment. (A)** Representative flow cytometric dot blot diagrams and histograms showing CD133-PE and CD44-APC surface pattern in exponentially grown SW480, SW620, and LS1034 cells kept under identical 2-D *in vitro* conditions. Antibodies and staining details are given in Table S1; the CD133 (AC133) fluorescence signal was enhanced by a two-step FASER series as previously described 23,33; **(B)** Engraftment rates of SW480, SW620, and LS1034 cells in NMRI nu/nu mice upon s.c. injection of defined single cell suspensions derived from exponentially grown monolayer cultures applied in limiting dilution approaches with 10 - 10,000 cells per mouse and injection site, respectively, n.d. - not determined; **(C)** Representative areas of H&E-stained 10 µm frozen sections from SW480, SW620, and LS1034 xenografts.

As prerequisite, we screened a panel of 20 established CRC cell lines for CD44 and CD133 (AC133) surface expression under absolutely identical, exponential 2-D culture conditions using our advanced staining protocol 33. Quite heterogeneous *in vitro* surface profiles were recorded (Figure S1A, Table S3), which is in line with heterogeneities seen in different, smaller CRC cell line panels 36,37. Three cell lines with distinct *in vitro* CD133/CD44 profiles (Figure 1A) were then selected for extended investigation: (i) SW480 cells comprise one main population highly positive for CD44 but lacking CD133 on the surface; (ii) SW620 cells, originated from the lymph node metastasis of the same patient, display all four technically possible subpopulations *in vitro,* but the CD44 surface level on the CD44^+^ fraction (68.1±2.3%) is lower than on SW480 cells (SI 16.9±2.5 vs. 34.4±21.0; Figure S1B); (iii) LS1034 is the only cell line model devoid of a clear CD44 surface presentation in culture. The cell fraction with CD133 fluorescence signals above isotype control in LS1034 2-D cultures amounts to 38.5±10.9%. However, the FC signal distribution as a whole shifted to the right (SI: 1.2±0.3) indicating that the entire LS1034 cell population is in principle slightly positive for CD133 (Figures 1 and S1, Table S3).

Limiting dilution experiments revealed engraftment of all three cell lines in NMRI(nu/nu) mice. TTR was highest for SW620, intermediate for LS1034, and lowest for SW480 cells (Figure 1B, Table S4A). Data did not imply any relation between *in vitro* CD133/CD44 pattern and engraftment, which is in agreement with findings in other CRC cell line models 23,33,36,37. SW480 and SW620 xenografts developed similar histomorphologies with poor differentiation, numerous mitotic figures, comparable stromal infiltration, and large necrotic areas with increasing tumor volume, whereas LS1034 tumors showed moderate differentiation with some glandular-like structures, lower mitotic index, and less necrosis (Figure 1C).

### Marker expression harmonizes in xenografts from CD133/CD44-sorted cell subfractions

**Figure 2:**
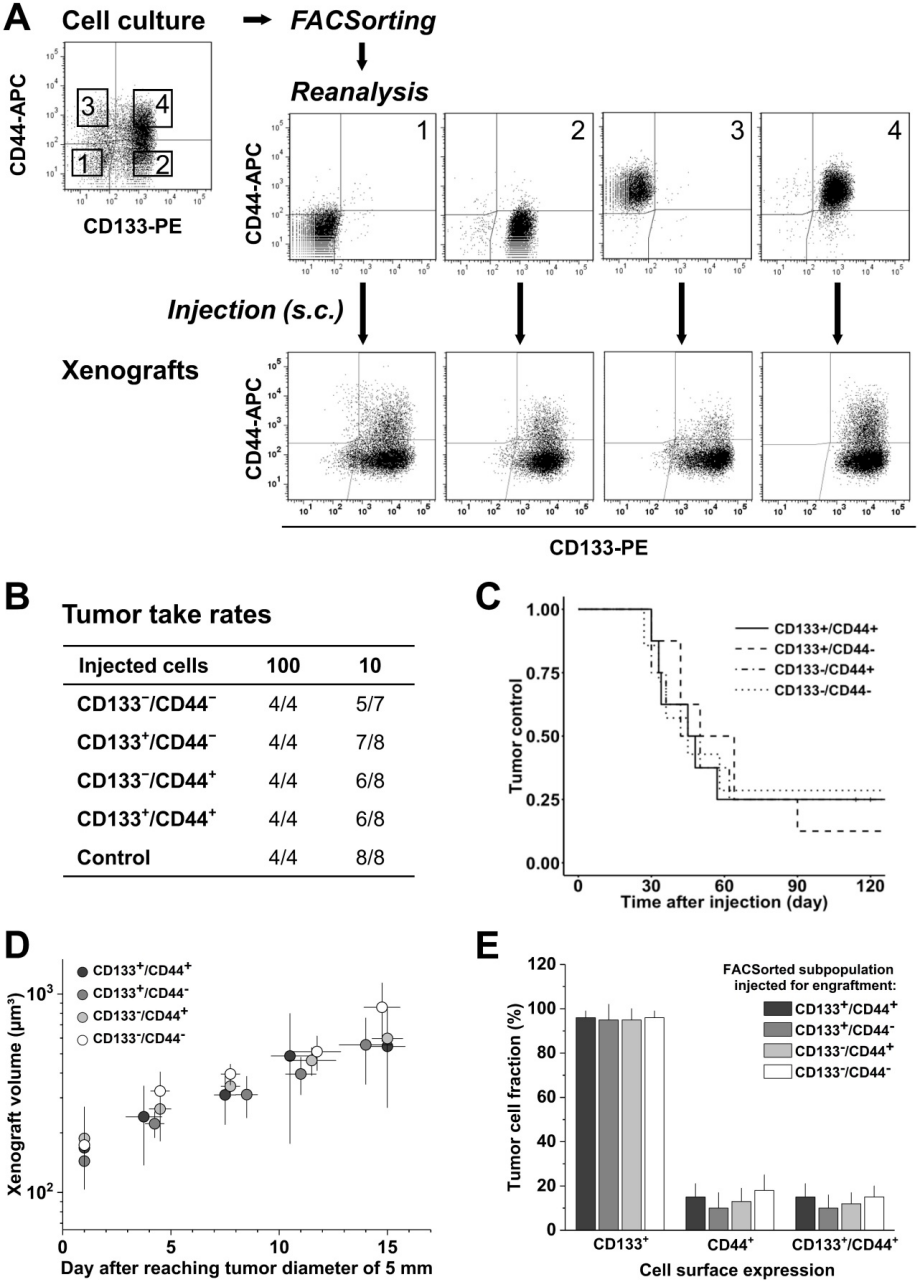
** CD133/CD44 surface expression in SW620 monolayer cells does not determine engraftment *in vivo*; CD133/CD44 pattern and population distributions in xenografts harmonize independent of the injected subpopulation. (A)** Representative flow cytometric dot blot diagrams of CD133-PE and CD44-APC surface pattern in SW620 cells before (cf. Figure 1) and after sorting for subcutaneous injection at limiting dilution (sort layout for subpopulations 1-4); bottom dot blots document representative CD133/CD44 pattern in cell suspensions derived from xenografts originated from the respective FACSorted subpopulation; **(B)** Engraftment rates of SW620 cell populations separated by FACS according to their *in vitro* CD133/CD44 surface pattern; control = stained cells processed (“run-through-sorter”) according to the subpopulations; **(C)** Tumor control as function of time after subcutaneous injections of 10 SW620 cells with different CD133/CD44 surface pattern (from B);** (D)** Normalized volume growth kinetics of xenografts derived from 100 SW620 cells with different CD133/CD44 surface pattern; **(E)** Distribution and proportion, respectively, of CD133^+^, CD44^+^, and CD133^+^/CD44^+^ SW620 cells (+SD) in xenografts originated from the different FACSorted *in vitro* SW620 subpopulations.

SW620 cells were sorted with high purity into their four subpopulations and initially characterized *in vitro*. Systematic analyses showed that the subpopulations do not differ in morphology or size, and they grow with an identical kinetics (Figure S2A). FC measurements of CD133/CD44 surface expression pattern revealed that the subpopulations partly redistribute after sorting over a period of 18 days in culture (Figure S2B, C), i.e., the proportion of CD44^+^ or CD133^+^ cells in the respective CD44^+^ or CD133^+^ sorted fractions decreased over time. However, this redistribution did not lead to a complete harmonization in the expression pattern of these cultures. We therefore subsequently injected similarly sorted SW620 subpopulations (Figure 2A) into NMRI(nu/nu) mice at cell numbers of 100 and 10 to verify if the CD44/CD133 surface expression is also irrelevant for subcutaneous SW620 cell engraftment.

Injection of 100 SW620 cells resulted in 100% engraftment, whereas 10 injected cells produced xenografts at frequencies of 71.4-87.5% (Figure 2B). No significant difference in TTR (Table S4B), TC, and normalized growth kinetics (Figure 2C, D) was observed upon injection of the different subpopulations, indicating that the tumorigenicity of SW620 cells is indeed independent of *in vitro* CD44/CD133 surface presentation. FC analyses of cell suspensions from xenografts further showed a relatively homogenous CD133 and CD44 pattern for all injected subfractions: CD133 was highly expressed on roughly all cells* in vivo*, i.e., the *in vitro* CD133^-^ subpopulations practically disappeared, whereas CD44 was clearly reduced and only detected on 10-18% of the SW620 xenograft cells (Figure 2A-E, cf. Figure 1A).

Engraftment relative to CD133/CD44 pattern was not studied for SW480 cells due to their uniform surface profile* in vitro*. However, a change in CD133/CD44 distribution in first generation xenografts versus monolayer culture qualitatively similar to the SW620 model was noted, i.e., CD133 surface expression increased while CD44 presentation decreased. There was a quantitative difference though: in SW480 xenografts, only a tumor cell fraction of 17±9% established CD133-positivity, whereas 64±13% still expressed CD44 (Figure S3).

**Figure 3:**
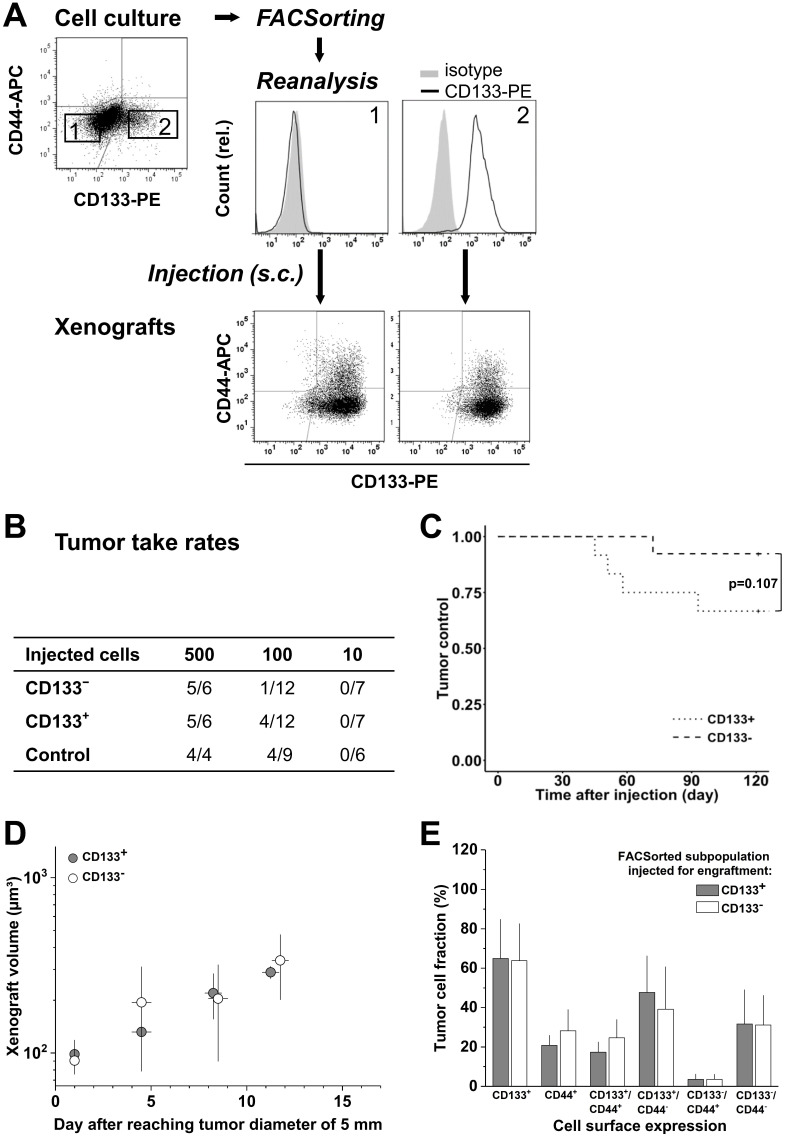
** CD133 pattern in LS1034 cells *in vitro* does not determine engraftment and *in vivo* behavior; CD133/CD44 cell surface profiles and distributions in LS1034 xenografts are consistent, with CD44 been newly expressed on a CD133^+^ subpopulation. (A**) Representative flow cytometric dot blot diagrams of CD133-PE and CD44-APC surface pattern in LS1034 cells before sorting (cf. Figure 1) and CD133 histograms of CD133^-^ (1) and CD133^+^ (2) subfractions after FACSorting before s.c. injection; bottom dot blots document representative CD133/CD44 pattern in cell suspensions prepared from xenografts originated from the respective injected subfractions; **(B)** Engraftment rates of LS1034 cell subfractions separated by FACS according to their *in vitro* CD133 surface expression; control = “run-through-sorter” (cf. Figure 2). **(C)** Tumor control as function of time after s.c. injection of 100 CD133^-^ or CD133^+^ LS1034 cells (according to B). **(D)** Normalized volume growth kinetics of xenografts derived from 500 CD133^-^ or CD133^+^ LS1034 cells; **(E)** Distribution and proportion, respectively, of CD133^+^, CD44^+^, and CD133/CD44 single or double negative and positive LS1034 cells (+SD) in xenografts originated from either CD133^+^ or CD133^-^ FACSorted *in vitro* cells.

Cultured LS1034 cells could be sorted with high purity into putative CD133^-^ and CD133^+^ fractions (Figure 3A), and were then injected for engraftment at limiting dilution. Ten cells did not engraft at all. In the 100-cell injections, the CD133^-^ fraction showed a slightly lower TTR and a marginally longer TC than CD133^+^ cells (Figure 3B, C). However, overall no significant difference was detected (Table S4C), and xenografts of >5 mm in diameter grew with comparable kinetics (Figure 3D). FC analyses revealed that CD133/CD44 surface expression pattern and distribution in LS1034 xenografts also harmonized independent of the injected subfraction. In this case, harmonization may relate to the assumption that CD133^-^ LS1034 cells represent the left margin of a CD133 weakly-positive population largely overlapping with the isotype. Most strikingly and adverse to the other models, CD44 was newly expressed on the surface of 21.8±9.3% LS1034 xenograft cells (Figure 3A,E).

### Biomarker profile in 1^st^ generation LS1034 xenografts defines secondary engraftment

**Figure 4:**
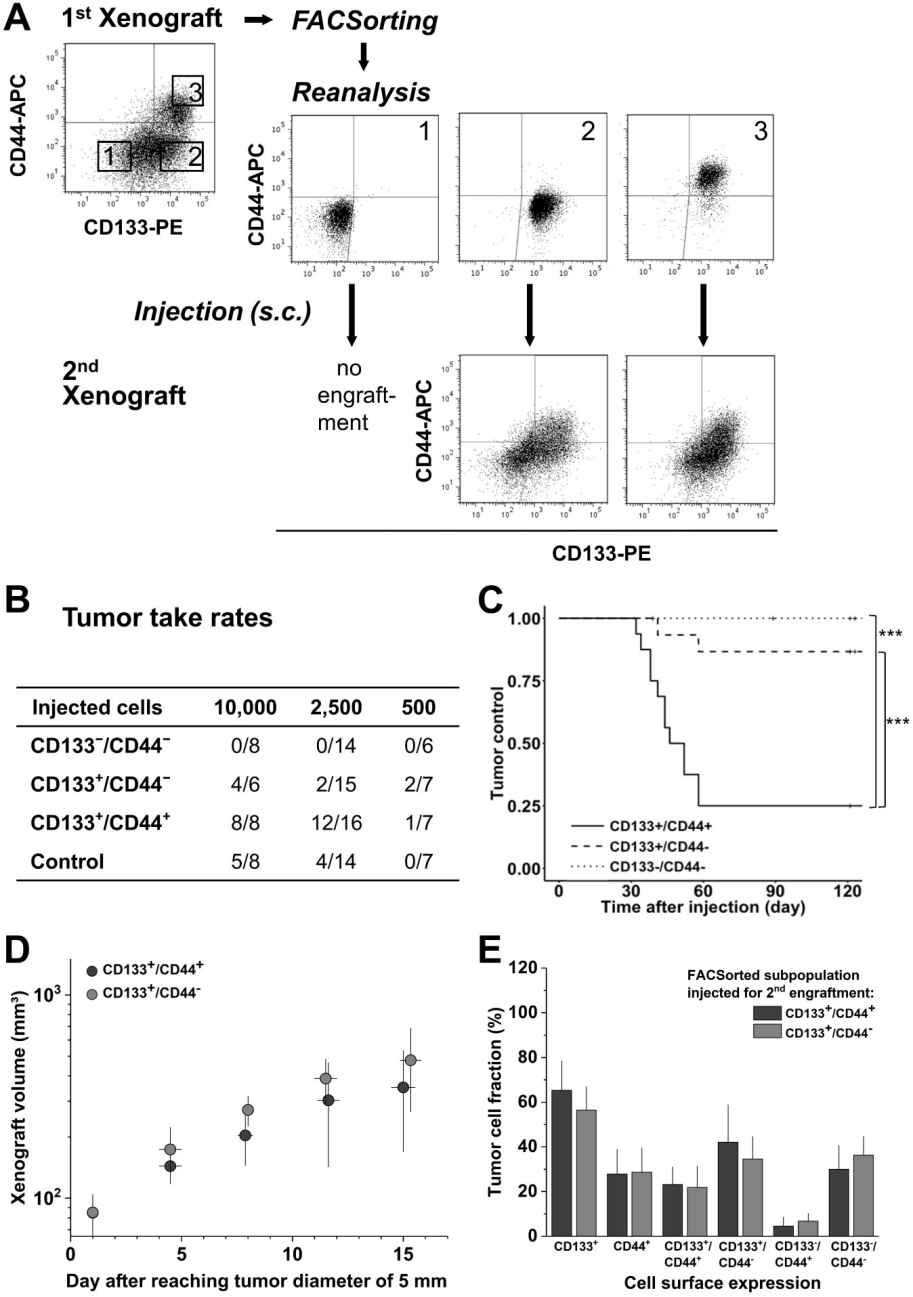
** CD133/CD44 pattern in LS1034 primary xenografts correlates with secondary engraftment. (A)** Representative flow cytometric dot blot diagrams of CD133-PE and CD44-APC surface pattern in suspensions of LS1034 xenograft cells before (cf. Figure 3) and after FACSorting and following secondary engraftment; **(B)** Engraftment rates of LS1034 xenograft cell populations separated by FACS according to their *in vivo* CD133/CD44 surface expression; control = “run-through-sorter” (cf. Figure 2). **(C)** Tumor control as function of time after s.c. injection of 2,500 FACSorted LS1034 cells originated from xenografts; *** p<0.001; **(D)** Normalized volume growth kinetics of secondary xenografts derived from 10,000 CD133^+^/CD44^-^ or CD133^+^/CD44^+^ LS1034 *in vivo* cells; **(E)** Distribution and proportion, respectively, of CD133^+^, CD44^+^ and CD133/CD44 single or double negative and positive LS1034 cells (+SD) in secondary xenografts originated from CD133^+^/CD44^-^ or CD133^+^/CD44^+^ cell populations isolated from primary LS1034 xenografts.

To address the relevance of *de novo* CD44 surface presentation* in vivo*, LS1034 xenografts were dissociated, stained, sorted according to their CD133/CD44 surface profile, and re-implanted for secondary engraftment (Figure 4A). In contrast to first generation engraftment, second generation TTR and TC differed significantly among subpopulations (Figure 4B, C & Table S4D), i.e., the CD133^+^/CD44^+^ subpopulation showed both highest engraftment rate and lowest TC, while the CD133^-^/CD44^-^ subpopulation had entirely lost tumorigenic potential. The engraftment of CD133^+^/CD44^-^ and control LS1034 cells was intermediate. Nonetheless, tumors that formed upon injection of 10,000 CD133^+^/CD44^-^ cells (4/8) showed comparable volume growth kinetics to those derived from the respective CD133^+^/CD44^+^ subpopulation (Figure 4D).

CD133/CD44 surface presentation in cell suspensions from secondary LS1034 xenografts resembled the expression profiles in first generation xenografts and revealed a harmonized biomarker pattern *in vivo* independent of the injected cell fraction (Figure 4A, E).

### Highly tumorigenic CD44^+^ LS1034 xenograft cells are located in well-oxygenated areas

It has been postulated that cancer stem-like cells are located or accumulate in particular microenvironments. In CRC, these so-called CSC niches have been hypothesized to comprise either vascularized 38-40 or hypoxic areas 41,42, while the invasive front has been considered as environmental niche for EMT-competent, metastasizing CSC 43. To elucidate the putative niche of highly tumorigenic CD44^+^ LS1034 cells, we extracted four first generation xenografts en bloc and prepared median frozen section for immunofluorescent detection of CD44^+^ cancer cells relative to (i) the CD31-positive endothelial cell vascular network and (ii) the pimonidazole hypoxic areas (pO_2_≤10 mmHg).

**Figure 5:**
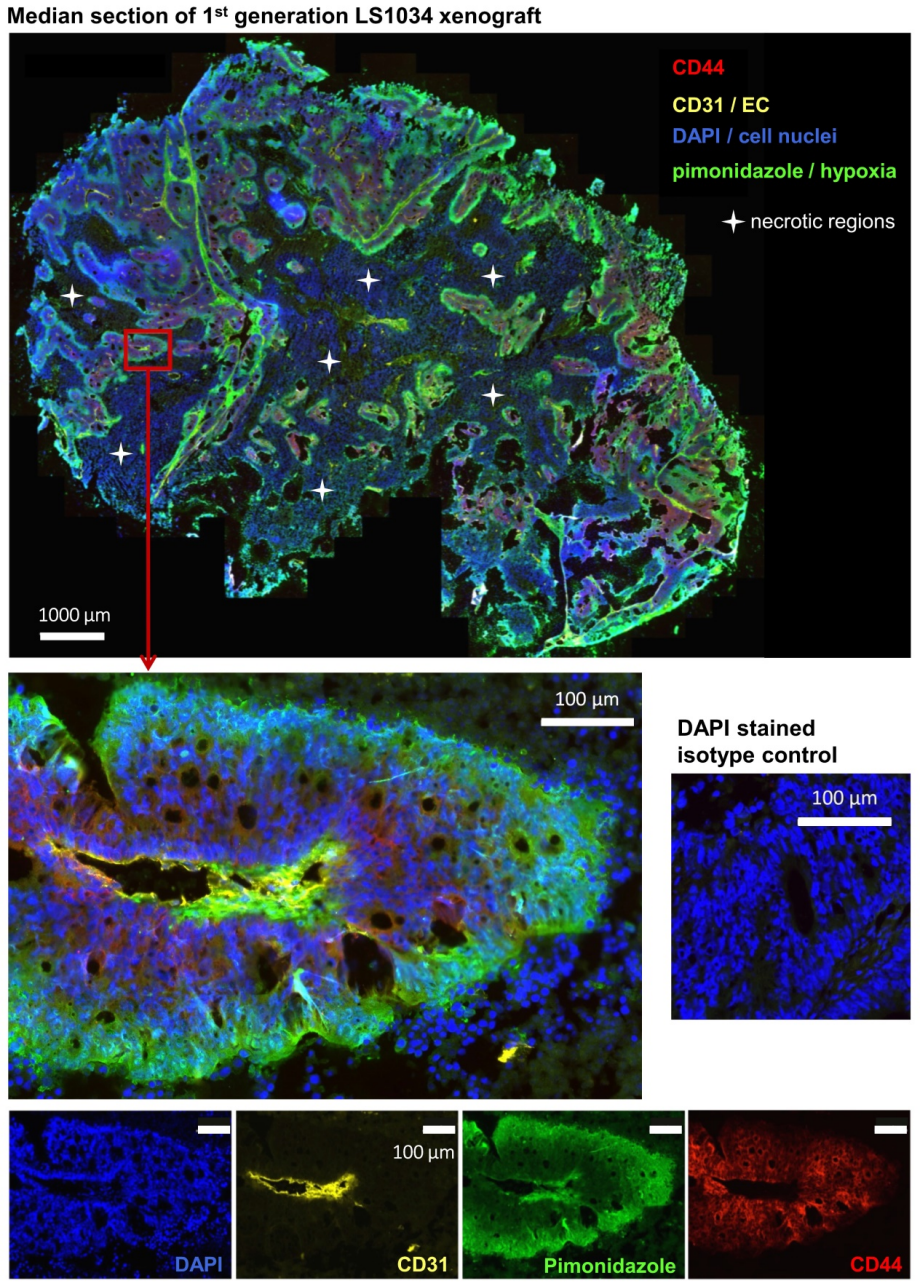
** CD44^+^ tumor cells are located in well-oxygenized but not in a proposed hypoxic cancer stem cell niche in LS1034 xenografts (see also Figure S4 A, B).** Median frozen section (10 µm) of an LS1034 xenografts co-stained for CD44, CD31 (endothelial cells), pimonidazole accumulation (hypoxia), and DAPI (nuclei) and imaged with a magnification of 200x; whole tumor section (stitched from >1,000 single images - top) and a selected region at higher magnification are displayed as four-channel overlays, while single channel images of the respective region are documented on the bottom.

As documented in Figures 5 and S4A, B, CD44^+^ LS1034 cells are located in well-oxygenated regions rather in proximity to vessels. No overlap between hypoxia and CD44-presenting cells was detected in any of the four LS1034 xenografts monitored, indicating that hypoxia can be excluded as niche for the highly tumorigenic CD44^+^ cells in this model.

### CD44 positivity in LS1034 xenograft cells is mainly due to up-regulation of transcription

**Figure 6:**
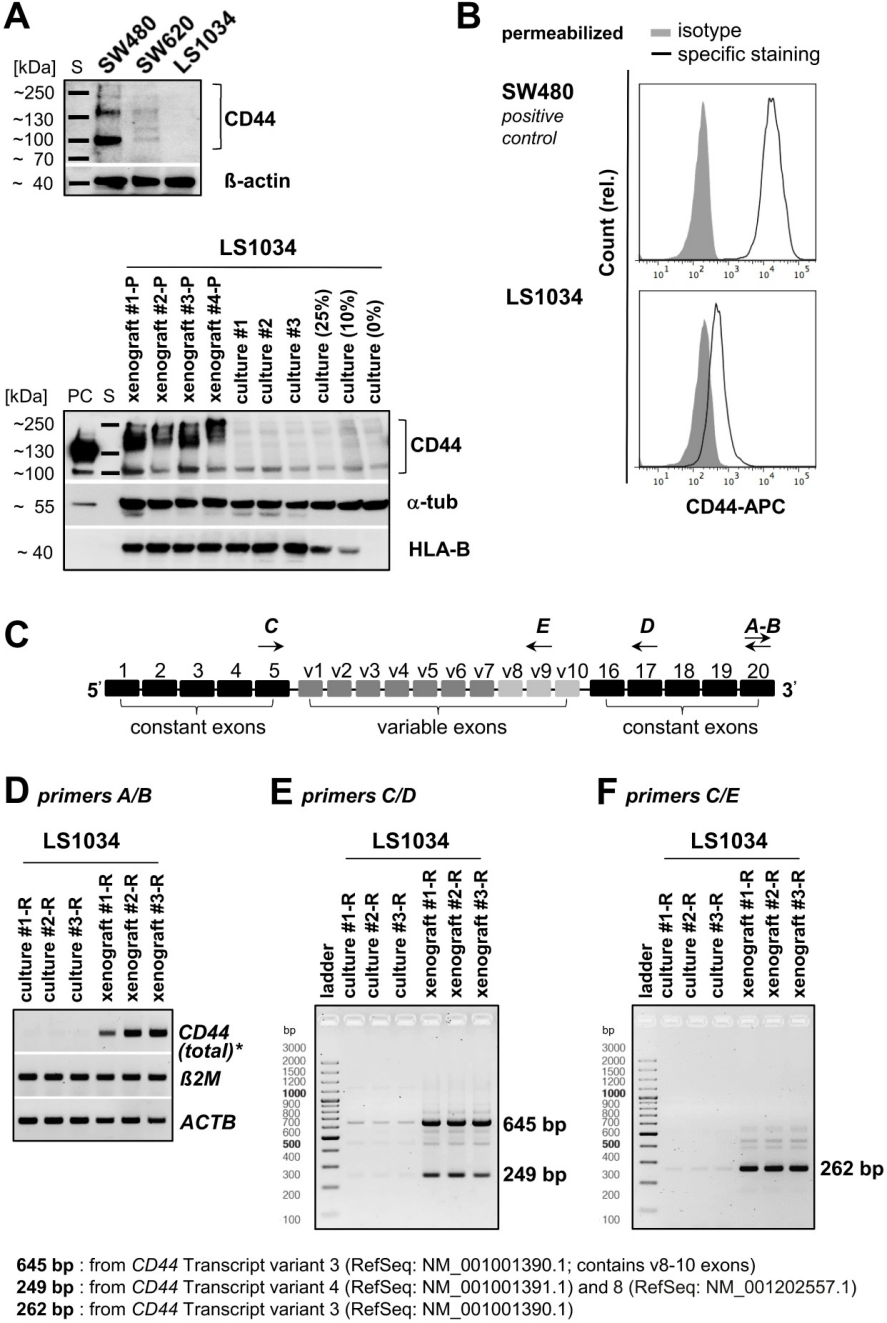
** CD44 enhancement in LS1034 xenografts is mainly due to up-regulation of *CD44*v8-10 expression. (A)** Upper panel: section of a representative Western blot according to Figure S5A (standard handling and illumination times, reducing conditions) to visualize the CD44 pattern in LS1034, SW620, and SW480 cells (40 µg protein loaded per lane). Lower panel: Western blot performed with similar protocol showing human-specific CD44 in protein lysates of four different LS1034 xenografts, three individual LS1034 monolayer culture samples, and lysates from cell mixtures containing 75%, 90%, or 100% mouse fibroblasts (= 25%, 10%, and 0% LS1034 cells); 50 µg protein loaded per lane (PC - positive control = 25 µg of an HT29 cell culture lysate); β-actin or α-tubulin were detected as protein loading controls (non-species specific), while HLA-B (MHC-I+HLA-B directed antibody) was displayed as human-specific control; **(B)** Representative flow cytometric histogram of CD44 labeling in permeabilized LS1034 cells (cf. Figure 1); SW480 cells served as positive control; **(C)** Scheme of human *CD44* gene structure, specific primer design, and representative RT-PCR analysis of **(D)** total, **(E)** isoform-specific, and **(F)** v9 exon-specific *CD44* mRNA expression in LS1034 cells *in vitro* and in xenografts; human *β-actin (ACTB)* and *B2M* mRNA levels were detected as reference. Note: Xenografts used for protein (-P) and RNA (-R) extraction were not identical as indicated by the respective name affix.

WB analyses, in principle, revealed the heterogeneity of CD44 protein profiles in CRC cell lines (Figures 6A and S5A). No signal was detected in LS1034 culture lysates, while SW480 monolayer cells produced three protein bands with different molecular masses and signal intensities. SW620 cells, which were inconsistently described in the literature as either CD44-negative 44 or CD44-positive 45, turned out to show no or only a very faint WB signal in spite of the clearly identified CD44^+^ subpopulations in FC. Therefore, we revealed CD44-positivity in SW620 cells by (i) using an alternative pan-αCD44 antibody (Figure S5B) and (ii) enhancing the WB sensitivity in a few experimental series. A critical extension (15-20×) of the WB illumination time allowed to detect a clear CD44 protein band at ~100-110 kDa in the SW620 cell lysates (Figure S5C), while LS1034 culture samples remained negative. Cell-line dependent differences in total CD44 protein levels *in vitro* thus correlate with the finding of CD44 surface presentation being negative in LS1034 and positive in SW620, but 3-times lower than in SW480 cells (% CD44^+^ cells × FC SI is 68.1×16.9 ≈ 1150 for SW620 vs. 99.8×34.4 ≈ 3,400 for SW480). Subsequently, we confirmed CD44 positivity in LS1034 xenografts; even with standard WB illumination times, CD44 could be identified in all lysates from four independent xenografts as broad band of high molecular mass indicative for a highly glycosylated CD44variant (Figure 6A).

Differences in CD44 protein pattern using pan-αCD44 antibodies may result from different gene expression levels, variable splicing, and/or various post-translational modifications. Hence, we next examined *CD44* gene expression profiles in LS1034 samples (Figure 6C-F). Total *CD44* gene expression level (*tv1-7*) was strongly enhanced in three independently engrafted LS1034 tumors compared to monolayer cultures (Figure 6D). In these experiments, we occasionally observed minor *CD44* gene expression in the LS1034 monolayer samples (Figure 6E, F), questioning the entire lack of CD44 protein in 2-D culture. As FC is often more sensitive than WB analysis, we performed CD44 labeling and FC measurements in permeabilized LS1034 cells and detected a slight right shift of the fluorescence signal relative to the respective isotype control, which is indicative for a very small amount of intracellular CD44 protein *in vitro* (Figure 6B). However, the hypothetical translocation of this CD44 to the cell surface is quantitatively negligible when it comes to the enhanced CD44 protein presentation on LS1034 cells under *in vivo* conditions, which clearly correlates with the upregulated *CD44* transcriptional activity.

### *CD44v8-10* exon expression is particularly enhanced in LS1034 xenograft cells

The *CD44* gene has 8 described mRNA transcript variants (NCBI GenBank®), which undergo complex alternative splicing (Figure S6A). Accordingly, we next identified the *CD44* gene transcripts which are selectively upregulated in the highly tumorigenic CD44^+^ cell subpopulation in LS1034 xenografts. We designed primer pairs covering the entire region of the variable exons (Figure 6C, Table S2) for qualitative RT-PCR to amplify all potentially transcribed *CD44* mRNAs.

Two strongly upregulated bands (249 bp and 645 bp) were identified in LS1034 xenograft cells as defined PCR products derived from particular *CD44* mRNA transcript isoforms (Figure 6E). These two bands were extracted and confirmed by sequence analysis (Figure S6B). The less pronounced PCR product at 249 bp might derive from either *CD44* mRNA *tv4* and/or *tv8* (Figures 6E and S6A), which both do not contain variable exons; *tv4* translates into standard CD44s protein, while *tv8* supposedly translates into a standard but cytoplasmically truncated, short-tail (st)CD44 protein isoform 46. CD44 gene analysis and the NCBI GenBank^®^ database 47 further indicated that the substantially strongest PCR product at 645 bp relates to *CD44* mRNA *tv3* containing the variable exons v8-10. To best prove this finding, we applied another primer pair particularly designed to amplify exon v9 and detected one prominently upregulated gene band at 262 bp (Figure 6F) deriving from *CD44* mRNA *tv3* (*CD44*v8-10) as confirmed by sequence analysis (Figure S6C). In summary, CD44v8-10 is clearly identified as the most abundant CD44 variant in LS1034 xenograft cells.

### *CD44v8-10* upregulation *in vivo* associates with transcriptional EMT markers

We next attempted to shed some light on the mechanism underlying *CD44*v8-10 overexpression in engrafted LS1034 tumors. Epithelial splicing regulatory proteins - ESRP1 and ESRP2 - were identified as key players for variable exon inclusion in CD44 isoforms 48,49. A strong downregulation of expression of ESRPs, in particular ESPR1, was reported during EMT (epithelial-mesenchymal transition), and has been associated with changes in cell morphology, loss of cell-cell interaction, and polarity, as well as elevated cell motility 50-52. By contrast, ectopic overexpression of ESRP1 seems to promote the switch from a mesenchymal to an epithelial cancer cell phenotype 51.

**Figure 7:**
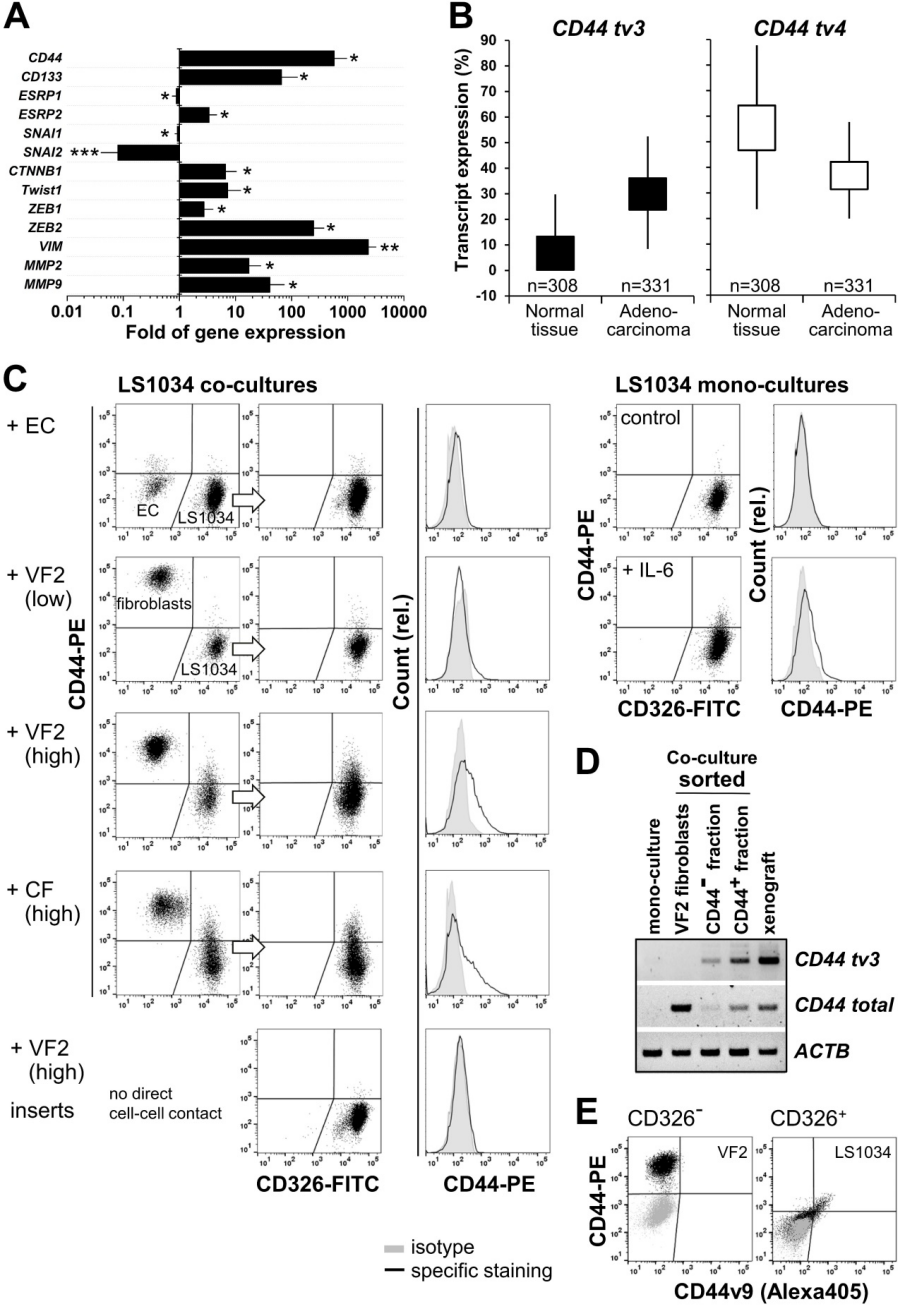
*CD44v8-10* upregulation *in LS1034 xenografts* associates with transcriptional EMT markers, can be triggered *in vitro* by interaction with stromal cells, and reflects the high expression level in primary colon adenocarcinoma tissues. (A) Fold of gene expression of CD44, CD133 as well as various EMT specific transcription factors and biomarkers in LS1034 xenografts versus cultured cells measured by q-PCR. Data normalized to *ACTB* gene expression are shown as means (±SD); * p<0.05; ** p≤0.01; *** p≤0.001; (B) Box plot comparing the *CD44 tv4* (st) and *CD44 tv3* (v8-10) transcript-specific expression (% of isoform) in colon adenocarcinoma TCGA (n=331) and primary normal colon epithelium GTEX (n=308) data (Welch's t-test - normal vs tumor tissue: *CD44 tv4*, p=3.952e-60, t=-18.87; *CD44 tv3,* p=7.748e-156, t=-36.12). Data were generated via the UCSC Xena platform (https://xenabrowser.net). (C) Representative flow cytometric dot blot diagrams and histograms documenting the CD44 surface presentation in membrane-intact LS1034 cells upon expose to IL-6 (10 ng/mL; mono-culture) or when co-cultured with human umbilical vein endothelial cells (HUVEC - EC), normal skin fibroblasts (VF2) or colon adenocarcinoma-derived fibroblasts (CF). LS1034 cells and CD44 highly positive fibroblasts were discriminated by CD326 labeling. Three examples of LS1034/VF2 co-cultures are documented to demonstrate the impact (i) of a higher fibroblast concentration (factor 1.7 -1.8) and (ii) of direct cell-cell versus paracrine interactions on the CD44 signal in LS1034. All acquired data are summarized in Table 1. (D) RT-PCR analysis of total and exon-specific *(tv3) CD44* mRNA expression in LS1034 *in vitro* cells grown in direct contact with fibroblasts. Cells were sorted according to their CD326/CD44 expression pattern: CD326^-^/CD44^+^ = VF2 fibroblasts, CD326^+^/CD44^-^ and CD326^+^/CD44^+^ = LS1034 cell fractions. LS1034 mono-culture and xenograft samples were analyzed in parallel as negative and positive controls. Primers A/B and C/D according to Figure 6D/F were applied and human *β-actin (ACTB)* mRNA served as reference. (E) Representative flow cytometric dot blot diagrams of CD44 (total) versus CD44v9 fluorescence signals on the surface of membrane-intact (PI-negative) VF2 fibroblasts (CD326^-^) and LS1034 cells (CD326^+^) after 4 days of co-culturing. The cell type-specific isotype controls are shown as overlay (grey).

In the LS1034 model, we found the engraftment-related upregulated expression of CD44 *tv3* to be paralleled by a reduced *ESPR1* expression. However, at the same time *ESRP2* mRNA levels were essentially enhanced (Figure 7A). Based on this seemingly contradictory observation, we also assessed the expression of well-known EMT transcription factors potentially participating in *CD44* regulation. Gene expression of *SNAI1* and especially *SNAI2* appeared to be downregulated *in vivo*. However, the transcript levels of *CTNNB1, TWIST1, ZEB1,* and in particular of* ZEB2* were significantly higher in LS1034 xenografts than in cultured cells. Similarly, mRNA expression of the established EMT markers* VIM, MMP2*, and *MMP9* was systematically and reproducibly enhanced upon LS1034 engraftment (Figure 7A). In summary, these results indicate that LS1034 CRC cells are more prone to switch from an epithelial to a mesenchymal phenotype under *in vivo* conditions due to an altered regulation and orchestration of EMT transcription factors. The scenario seems to be linked to the enhanced expression of *CD44 tv3* and CD44v8-10, respectively, probably via the downregulation of *ESRP1* expression.

### *CD44v8-10* variant is upregulated in primary CRC tissues

To better assess the potential of the *CD44 tv3* positive LS1034 *in vivo* model and the related findings, we performed in silico bioinformatics analysis of transcript-specific *CD44* gene expression (distribution of whole isoforms values - % of isoforms) in CRC tissues compared to normal colon epithelia based on the TCGA TARGET GTEx dataset. In normal and tumor colon tissues, *CD44 tv3* (v8-10) and *CD44 tv4* (st) are the most strongly expressed *CD44* mRNA transcripts among all *CD44v* isoforms (Figure 7B, Figure S7A/B). However, while *CD44 tv4* (CD44s) expression is significantly lower in CRC than primary normal colon tissues (p≤0.001), *CD44 tv3* (CD44v8-10) shows the opposite with enhanced expression in the colon adenocarcinomas samples (Figure 7B).

### Stromal cells but not milieu conditions trigger CD44 upregulation in LS1034 cells

We finally aimed at modifying the *in vitro* culture conditions to identify a unique biological modifier to induce CD44 in LS1034 cultures for reflecting the *in vivo* situation. In line with the observation of CD44^+^ LS1034 cells to not be located in the hypoxic regions, CD44 protein expression could not be induced in LS1034 cultures by various pathophysiological stress conditions such as (tissue) acidosis or lactate accumulation, although some of these conditions were found to enhance CD44 in SW480 and SW620 cell cultures (e.g., Figure S5C). Our experimental series to mimic selective aspects of the *in vivo* situation included the use of diverse serum-free and serum-conditioned classical and stem cell media, various 2-D and 3-D culture conditions, as well as the culturing on diverse ECM-coated surfaces and/or under tissue normoxia. None of these environmental constraints induced CD44 surface presentation (Table 1). Only when adding 10 ng/mL IL-6 to the supernatant, a slight shift in the CD44 signal was observed with 2-3% of the LS1034 cells showing CD44 fluorescence intensities above background (Fig. 7C). This effect could not be enhanced by increasing IL-6 concentrations (Table 1). A more pronounced CD44 induction was only seen when LS1034 cells were co-cultured with fibroblasts. The addition of HUVEC to the fibroblasts at a 1:15 ratio, according to a co-culture angiogenesis assay leading to tubule formation, was not supportive. Likewise, exclusive co-culturing with HUVEC, even at very high seeding densities of 4.3×10^4^ EC per cm^2^, only marginally affected the CD44 expression in LS1034 cells.

The impact of fibroblasts became stronger at higher densities, i.e., a CD44^+^ LS1034 fraction of about 10% was observed after 4 days of co-culturing when (1.2-1.3)×10^4^ fibroblasts per cm² culture surface were seeded 24 h prior to the addition of the same number of tumor cells (Figure 7C, Table 1). We applied both normal skin (VF2) and colon adenocarcinoma (CF)-derived fibroblast and found them to be similarly effective. The main trigger for CD44 upregulation in LS1034 cells are direct fibroblast-tumor cell interactions, because non-contact co-culture approaches in 6-well plates with inserts were ineffective.

RT-PCR analysis of total and v9 exon-specific *CD44* mRNA expression in LS1034 and VF2 fibroblasts sorted after 4 days in confrontation culture revealed a fibroblast-triggered upregulation of *CD44 tv3* translating into CD44v8-10 in the LS1034 cells; the CD44 highly-positive fibroblasts did not express this transcript variant (Figure 7D). A selective CD44v9-directed antibody further confirmed that the positive shift in the CD44 fluorescence signal observed by FC relates to enhanced CD44v8-10 surface presentation in the co-cultured LS1034 cells. In contrast, no CD44v9 signal beyond background autofluorescence was detected in the CD44-positive fibroblasts (Figure 7E).

## Discussion

Critical reflection of the booming literature addressing CSC in solid cancers, including gastrointestinal malignancies, implies that hardly any other topic in cancer research has been more prone to data mis- or overinterpretation in the past decade. Problems and controversies still arise from (i) inconsistent semantics and terminology in the field, (ii) putative, functionally non-linked or poorly-defined surrogate markers despite their potential prognostic and predictive value - this applies to many CD surface markers, e.g. the transmembrane glycoprotein CD133 studied herein 53,54, and (iii) splice variants and posttranslational modifications of proposed CSC surrogate markers that are often not sufficiently discriminated in preclinical and clinical studies such as CD44, our second marker of interest 55.

Further hurdles are due to (iv) detection methodologies with different sensitivities and specificities as well as misleading definitions of marker-positive and -negative subpopulations as pointed out earlier 23,33,53 and also in the present study.

Limitations of functional CSC assays must also be carefully considered. The *in vivo* limiting dilution approach with consecutive transplantations is still the state-of-art gold standard to identify CSC and CSC-enriched populations, respectively, from human solid tumors 55,56. However, it remains unclear whether we primarily select for the functional advantage of some cancer (stem-like) cells to more easily adhere or survive in the environment of a particular recipient and/or injection site. Nonetheless, it is functionally highly informative to compare the sustained engraftment capacity of different human cancer cells and subpopulations, respectively, in a given animal model and engraftment site when using defined and reproducible operating procedures.

Last but not least, cancer researchers have long appreciated that high-passage cancer cell lines do not entirely recapitulate human malignant disease 57. Ben-David et al. 58 recently revealed the extent to which individual established tumor cell lines are heterogeneous, resulting from both clonal dynamics of pre-existing sub-clones and continuous instability leading to diversification during long-term culturing. These findings, together with the numerous controversial observations related to CSC surrogate markers and engraftment in cell lines clearly fueled the debate on the necessity for primary, patient-derived low-passage tumor cells for CSC-related research 59. On the other hand, besides and complementary to the Cancer Cell Line Encyclopedia project 60, comparative exosome-capture and transcriptome sequencing combined with microsatellite instability, SNP microarray, and promoter methylation analyses in 70 CRC cell lines confirmed a high level of overlap in the genomic landscape of primary CRC in the TCGA and human CRC cell lines 61. Despite some differences, globally similar genetic alterations referring to DNA copy number as well as genome-wide and driver gene mutation profiles were found. Other studies showed gene expression and proteome profiles in CRC cell line models to broadly represent those of primary tumors 21,62. Besides fundamental contemporary technological progress, e.g., in the use of genetically engineered mouse (GEM) models or patient-derived xenograft (PDX) models and organoids 63-66, cancer cell lines will remain a central tool in cancer research and treatment for the next decades 55,67. Accordingly, the extent to which established CRC cell lines can reflect CSC and non-CSC phenotypes and functions need to be further explored with utmost care.

In the present study, we found the CRC cell line LS1034 to yield intermediate (to poorly) differentiated subcutaneous xenografts and fulfill the main criteria of cancer stemness concerning cellular heterogeneity, engraftment capacity, and development of tumorigenic and non-tumorigenic progeny. We discovered a pivotal role of the *in vivo* environment for developing CD133/CD44 surface expression profiles in LS1034 subpopulations that correlate with tumorigenic potential as expected for putative surrogate markers. The expression of the same molecules *in vitro* was unrelated to engraftment not only for LS1034 but also SW620 cells. Evidence in the LS1034 cell line model suggests stromal fibroblasts as critical players in this scenario, an observation that might well explain some of the controversies in the literature related to CD marker expression and tumorigenicity in established CRC cell lines and primary cancer cells. In principle, the data indicate that neither CD133 nor CD44 are *per se* CSC markers as their expression seems highly plastic in CRC cells, and their usefulness as surrogate markers for engraftment capacity appears to be environmentally conditioned. The modulators and drivers of spatiotemporal and locoregional cancer (stem) cell plasticity still remain an unresolved issue in this context. Indeed, the reciprocal relationship between CSC and the microenvironment is still insufficiently understood 4,5,38-40,68. The highly tumorigenic LS1034 xenograft cells newly express CD44 *in vivo* and could be localized in the well-oxygenated areas in proximity to perfused vessels. This finding does not support a hypoxic but is in line with a previously proposed perivascular niche for CRC CSC 38-40.

Initially, we could not identify a simple trigger for inducing CD44 on the surface of LS1034 cells *in vitro* - even by exposing the cells to numerous diverse 2-D and 3-D environmental conditions. Myofibroblast-secreted factors were proposed earlier to restore a CSC phenotype in more differentiated colon cancer cells both *in vitro* and *in vivo,* suggesting that CRC stemness is in part orchestrated by stromal compartments 69. One of the multifunctional, protumoral factors of interest secreted from myofibroblasts and cancer-associated-fibroblasts (CAF), respectively, is IL-6 (e.g. 70,71), which indeed slightly enhanced the CD44 surface signal in LS1034 cell cultures. However, direct co-culturing of LS1034 cells with fibroblasts was much more effective in inducing tumor cell surface CD44 indicating that IL-6 is not the central in this scenario. This is underlined by two observations: (i) the lack of IL-6 concentration dependency of CD44 induction and (ii) the comparable efficacy to trigger tumor cell surface CD44v8-10 of CAF and normal foreskin fibroblasts, with the latter usually producing less IL-6 72. Contact-free co-culturing turned out to be entirely ineffective suggesting that paracrine factors released from the fibroblasts at distance are unlikely to play a major role in the CD44 induction in cancer cells. A recent study reported that CAF can induce a CSC-like phenotype and inherent resistance to chemotherapy in CRC cells through exosomal transfer of long non-coding RNAs, i.e., H19 73. Here, H19 was shown to activate the β-catenin pathway and upregulate the expression of downstream genes including CD44 by acting as a competing endogenous RNA sponge for miR-141 73, which is a member of the miR-200 family of microRNAs known to be involved in the regulation of EMT in CRC 74. Follow-up studies shall, therefore, address the mechanistic relevance of direct cell-cell contacts and the putative contribution of exosomal transfer of materials in the regulation of CD44, and in particular CD44v8-10, in the LS1034 CRC *in vitro/in vivo* model.

Exploring the microenvironmental impact on the expression pattern of diverse CD44 variants in CRC remains a major challenge. CD44s has been reported earlier to appear throughout the adenoma-carcinoma sequence 75, and CD44* tv* that translate into standard CD44 proteins were also found to be more abundant in LS1034 xenografts than in cultured cells. However, the strongly enhanced, quantitatively more relevant expression of *CD44 tv3* as the prime transcript related to CD44 *de novo* presentation *in vivo* that correlates with highest tumorigenicity merits particular attention. At least ten exons (v1-v10) can be alternatively spliced in various combinations, thereby generating a mixture of variant isoforms (CD44v) with various molecular weights, diverse interaction partners, and different functions, some of which might be integral to stemness and plasticity in epithelial malignancies 9,55,75-79. In this context, CD44v6 is often regarded as the main CD44v of interest as it has been associated with a malignant phenotype and poor outcome in CRC patients in the majority of scientific reports, and it is also suggested as molecular imaging target for sporadic and high-risk Lynch adenomas 18,80,81. Furthermore, CD44v6 is considered a common marker of constitutive and reprogrammed CSC driving CRC metastasis 18,19,82, and is currently studied as a target in phase I clinical trials for epithelial and colorectal cancers, respectively 18,19,83. We could not identify CD44v6 but instead found an essentially induced *CD44 tv3* translating into CD44v8-10 protein in the LS1034 xenografts.

Notably, a large amount of data from The Cancer Genome Atlas (TCGA) is being generated and analyzed to reveal different targeting approaches 84. In silico datasets and our results confirm that the CD44v8-10 splice variant is a major CD44 isoform in CRC cancer, and its expression in the tumors is much higher than that of other variants. CD44v8-10 has indeed been described to be overexpressed and correlating with metastasis in several human epithelial malignancies, including colon and gastric cancers 85-88. Some of these studies further propose CD44v8-10 as predictive marker for recurrence and poor survival in CRC and gastric cancer patients, and imply an association with a CSC-like phenotype in these entities. However, little is known about the functions of this isoform. So far, exon v9 containing isoforms were shown to interfere with death receptor FAS (CD95), thereby triggering apoptosis resistance 89. CD44v8-10 also seems to stabilize the cell membrane transporter xCT, which constitutes the limiting step of glutathione synthesis as key element in the cell's defense against reactive oxygen species (ROS) 89. The proposed mechanisms are likely to support CRC cell survival and therapy resistance and would thus also be beneficial for CRC CSC with detrimental consequences for patients. Accordingly, Ju et al. have demonstrated a high abundance of CD44v8-10 in stem-like CRC side population cells and their functional regulation by glutathione-mediated reduction in cellular ROS levels via the CD44v-xCT axis 90. In addition, a therapeutic approach to eliminate the chemotherapeutically resistant side population by disrupting the redox status has been tested *in vivo* with promising results, i.e., the application of the GSH-conjugating compound phenethyl isothiocyanate (PEITC). Other treatment options to impair the CRC CSC defense mechanisms against ROS, such as the targeting of the CD44v-xCT system, have been discussed by the authors, and miR-1297 was proposed as diagnostic and putative predictive marker in CRC as it directly targeted xCT 90. Further identification and study of CD44 isoforms and their interdependences with the microenvironment and cellular metabolism in CRC models, such as the LS1034 *in vitro/in vivo* system, are thus of great therapeutic relevance and the pre-requisite for the design of new combinatorial treatment concepts.

Sequence analysis of the amplified *CD44 tv3* in LS1034 xenograft cells evinced a monoallelic mutation in *exon v8* (c.689T>C [p.Ile230Thr]; Figure S6B/C). The identified transition is located in the proposed stem cell region of the extracellular domain which interacts with external ligands and the microenvironment 77 and has already been described in the NCBI database of single nucleotide polymorphisms (https://www.ncbi.nlm.nih.gov/SNP) and the Unitpot database (UniProtKB-P16070). The relevance of this genetic modification is unknown. The LS1034 model with its inducible CD44v8-10 expression triggered by stromal fibroblasts in a particularly tumorigenic subpopulation seems to be a highly valuable tool to gain mechanistic insight into the functional role of this CD44 variant in colon cancer progression. In this context, our study showed that *CD44 tv3* expression not only positively correlates with LS1034 engraftment but also appears to be directly or indirectly linked to the epithelial-mesenchymal transition (EMT) machinery. Based on the EMT marker gene expression profiles in LS1034 xenografts, we propose the EMT-associated transcription factors *SNAI2* and *ZEB2* to be most relevant to the upregulation of *CD44* expression and a negative regulatory role for ESRP1. Extended studies are envisioned to prove causality, gain functional insight, and evaluate motility and invasive behavior as well as metastatic potential of LS1034 cells relative to their CD44v8-10 phenotype.

Phenotypic and CD surface expression dynamics were clearly observed for *in vivo* LS1034 cell fractions with intermediate (CD133^+^/CD44^-^) and high engraftment rates (CD133^+^/CD44^+^), which can both develop from each other and also give rise to the non-tumorigenic progeny. However, plasticity in CD44 expression and engraftment capacity were entirely lost in an LS1034 subfraction lacking both CD44 and CD133* in vivo.* This is indicative for a non-flexible phenotype of this non-tumorigenic progeny exclusively manifested *in vivo*. Whether it can be induced *in vitro* by microenvironmental factors - according to the CD44v8-10 induction seen in LS1034 cells in co-culture with fibroblasts - remains to be elucidated. The phenomenon should be further explored because biomolecules stimulating this one-way phenotypic shift may have enormous therapeutic potential. Previous studies showing not only a hierarchical organization of CRC but also CSC dynamics imply a plastic subpopulation of Lgr5(+) CSC 10,11. As a consequence, we propose to monitor Lgr5 and additional putative stem cell markers in the LS1034 model and its CD133/CD44-defined subpopulations. We further suggest to evaluate our findings in other cell line models with intermediate or low tumorigenicity such as SW1222, which were documented earlier to contain only small amounts of self-renewing CD44^+^(CD24^+^) cells *in vitro* and to produce xenografts resembling well-differentiated primary human lumen-forming CRC 91.

## Conclusions

Our data unequivocally show that CD surface markers can be invalid definitions for tumorigenicity and CRC stemness when ignoring their environmentally-driven plasticity and dynamics. The LS1034 CRC cell line turned out to be a unique model for functional studies due to (i) the irreversible loss of engraftment potential accompanying CD133-negativity *in vivo* but not *in vitro*, and (ii) the *de novo* expression of CD44, and in particular of CD44v8-10, correlating with highest tumorigenicity *in vivo*. CD44 surface presentation in LS1034 cell *in vitro* could be critically induced only by interaction with stromal fibroblasts but not by many other external factors, including 3-D stem cell conditions. Perivascular areas in CRC often comprise cancer-associated fibroblasts. This might explain literature data showing CD133/CD44-positive CSC in primary CRC to be located in a so-called perivascular niche as well as our own observation of CD44-positivity in CRC cells in the non-hypoxic area in proximity to vessels. Hence, the identification and further detailed study of both intercellular communication and intracellular signaling pathways that causally relate to the selective CD44v8-10 upregulation triggered by the fibroblast-tumor cell interaction in the LS1034 *in vitro/in vivo* model is of utmost interest. Treatment options that either drive cancer cells into a permanent non-tumorigenic state or those preventing the shift to a CSC tumorigenic behavior may ultimately improve the outcome of CRC patients even at advanced stages. Further unraveling the mutual interrelation between microenvironment, cancer cell phenotype, surface marker expression, and cancer stemness in LS1034 and other CRC cell models will, therefore, allow for improvements in current *in vitro* therapy test platforms and contribute to the development of novel combinatorial anti-cancer treatment strategies targeting a CSC supportive microenvironment.

## Supplementary Material

Supplementary figures and tables.

## Figures and Tables

**Table 1:** Environmental factors and conditions tested either negative (-) or positive (+) for induction of CD44 surface presentation in LS1034 cells *in vitro.* The fractions of cells with CD44 fluorescence signals higher than background are given for (+) samples; mean ± SD for N≥3 or individual values for N<3 independent experiments are listed.

Culture vessel	Physiological / pathophysiological modification in				N^§^	CD44induction
	Surface coating	Culture format	Medium*	Milieu/Environment^†^		
Various	Original	2-D exp. / non-confluent	DMEM+		>25	Control
T25 flask	original	**2-D confluent**	DMEM+		2	-
T25 flask	original	**2-D post-confluent**	DMEM+		2	-
6-well plate	**poly-D-lysin**	2-D exp. / non-confluent	DMEM+		3	-
6-well plate	**fibronectin**	2-D exp. / non-confluent	DMEM+		3	-
6-well plate	**laminin**	2-D exp. / non-confluent	DMEM+		3	-
6-well plate	**collagen type I**	2-D exp. / non-confluent	DMEM+		3	-
6-well plate	**collagen type IV**	2-D exp. / non-confluent	DMEM+		3	-
6-well plate	**hyaluronan (0.1 mg/mL) mg/ml)**	2-D exp. / non-confluent	DMEM+		2	-
6-well plate	**hyaluronan (0.2 mg/mL)**	2-D exp. / non-confluent	DMEM+		2	-
6-well plate	**hyaluronan (0.5 mg/mL)**	2-D exp. / non-confluent	DMEM+		2	-
6-well plate	**hyaluronan (1.0 mg/mL)**	2-D exp. / non-confluent	DMEM+		2	-
6-well plate	original	2-D exp. / non-confluent	DMEM+	**4% O_2_**	3	-
6-well plate	**poly-D-lysin**	2-D exp. / non-confluent	DMEM+	**4% O_2_**	3	-
6-well plate	**fibronectin**	2-D exp. / non-confluent	DMEM+	**4% O_2_**	3	-
6-well plate	**laminin**	2-D exp. / non-confluent	DMEM+	**4% O_2_**	3	-
6-well plate	**collagen type I**	2-D exp. / non-confluent	DMEM+	**4% O_2_**	3	-
6-well plate	**collagen type IV**	2-D exp. / non-confluent	DMEM+	**4% O_2_**	3	-
10 cm dish	original	2-D exp. / non-confluent	DMEM+	**pH 6.9**	2	-
10 cm dish	original	2-D exp. / non-confluent	DMEM+	**pH 6.4**	2	-
10 cm dish	original	2-D exp. / non-confluent	DMEM+	**10 mM lactate**	2	-
10 cm dish	original	2-D exp. / non-confluent	DMEM+	**10 mM lactate, pH 6.9**	2	-
10 cm dish	original	2-D exp. / non-confluent	DMEM+	**20 mM lactate**	2	-
10 cm dish	original	2-D exp. / non-confluent	DMEM+	**20 mM lactate, pH 6.4**	2	-
10 cm dish	original	2-D exp. / non-confluent	**DMEM-**		2	-
10 cm dish	original	2-D exp. / non-confluent	**SC1+**		2	-
10 cm dish	original	2-D exp. / non-confluent	**SC1-**		2	-
6-well plate	**hyaluronan (HYS020)**	**3-D / spheres^‡^**	DMEM+		2	-
6-well plate	**ultra-low attachment**	**3-D / spheres^‡^**	**SC2-**		3	-
96-well plate	**agarose**	**3-D / clusters^‡^**	**SC2-**		3	-
96-well plate	**agarose**	**3-D / spheroids (~ 400 µm)**	DMEM+		3	-
96-well plate	**agarose**	**3-D / spheroids (600-650 µm)**	DMEM+		6	-
96-well plate	**agarose**	**3-D / spheroids (>800 µm)**	DMEM+		3	-
6-well plate	**original + insert**	**co-culture (F, non-contact)**	DMEM+	**1.2×10^5^ - 5×10^5^ VF2^**	3	-
6-well plate	original	2-D exp. / non-confluent	DMEM+	**IL-6 (10 ng/mL)**	4	2.3 ± 0.2
6-well plate	original	2-D exp. / non-confluent	DMEM+	**IL-6 (50 ng/mL)**	4	2.7 ± 0.1
6-well plate	original	2-D exp. / non-confluent	DMEM+	**IL-6 (100 ng/mL)**	4	2.7 ± 0.4
6-well plate	original	**co-culture (EC)**	DMEM+	**2×10^5^ EC^**	2	1.1/1.2
6-well plate	original	**co-culture (EC)**	DMEM+	**4×10^5^ EC^**	2	1.5/2.1
6-well plate	original	**co-culture (F/EC)**	DMEM+	**0.7×10^5^ VF2 + 5×10^3^ EC^**	2	3.3/3.8
6-well plate	original	**co-culture (F/EC)**	DMEM+	**0.7×10^5^ CF + 5×10^3^ EC^**	2	2.8/3.3
6-well plate	original	**co-culture (F/EC)**	**EGM2**	**0.7×10^5^ VF2 + 5×10^3^ EC^**	2	2.0/3.3
6-well plate	original	**co-culture (F/EC)**	**EGM2**	**0.7×10^5^ CF + 5×10^3^ EC^**	2	2.5/2.6
6-well plate	original	**co-culture (F)**	DMEM+	**0.7×10^5^ VF2^**	2	3.5/4.3
6-well plate	original	**co-culture (F)**	DMEM+	**0.7×10^5^ CF^**	2	3.4/4.0
6-well plate	original	**co-culture (F)**	DMEM+	**1.2×10^5^ VF2^**	4	11.2 ± 0.5
6-well plate	original	**co-culture (F)**	DMEM+	**1.2×10^5^ CF^**	4	10.1.± 0.4

*DMEM: standard medium; SC1/SC2: stem cell medium 1 & 2; +/- : with/without 10% FCS; EGM2: suppl. EC growth medium; †standard: 18-19% O_2_, pH 7.2-7.4; §N: number of independent experiments; ‡spheres were <100 µm in size as opposed to larger spheroids; the term *cluster* describes the formation of small, flat 3-D aggregates; ^number of stromal fibroblasts (F) or endothelial cells (EC) seeded per well 24 h before adding 1.2 × 10^5^ LS1034 cells;VF2: normal foreskin fibroblasts; CF: colon carcinoma-derived fibroblasts; EC: human umbilical vein endothelial cells (HUVEC).

## Abbreviations

2-D: two-dimensional
3-D: three-dimensional
Ab: antibody
ACTB: beta (β)-actin
ALDH: aldehyde dehydrogenases
APC: allophycocyanin
ATCC: American Type of Culture Collection (USA)
BCA: bicinchoninic acid
bp: base pair(s)
B2M: beta (β)-microglobulin
CAF/CF: cancer-associated fibroblasts/colon cancer-derived fibroblasts
CD: cluster of differentiation
CD44s: CD44 standard isoform
CD44v: CD44 variant isoform
CRC: colorectal cancer/carcinoma
CSC/TPC/TIC: cancer stem(-like) / tumor propagating / tumor-initiating cell(s)
CTNNB1: catenin beta (β)-1
DAPI: 4′,6-diamidino-2-phenylindole
DMEM: Dulbecco's modified Eagle's medium
EC: endothelial cell(s)
EDTA: ethylenediaminetetraacetic acid
EGF: epidermal growth factor
EGM-2: endothelial cell growth medium 2
EMT: epithelial-mesenchymal transition
ESRP1/ESPR2: epithelial splicing regulatory protein 1 or 2
F: fibroblast(s)
FACS: fluorescence-activated cell sorting
FASER: fluorescence amplification by sequential employment of reagents
FC: flow cytometry
FCS: fetal calf serum
FGF: fibroblast growth factor
FITC: fluorescein isothiocyanate
GEM: genetically engineered mouse
HEPES: 4-(2-hydroxyethyl)-1-piperazineethanesulfonic acid
H&E: hematoxylin and eosin
HLA: human leukocyte antigen
HUVEC: human umbilical vein endothelial cell(s)
IL-6: interleukin-6
MEBM: mammary epithelial cell basal medium
MES: 2-(N-morpholino)ethanesulfonic acid
MMP: matrix metalloproteinase
PBS: phosphate-buffered saline
PC: positive control
PDX: patient-derived xenograft
PE: phycoerythrin
PFA: paraformaldehyde
PI: propidium iodide
PMFS: phenylmethylsulfonyl fluoride
RIPA: radioimmunoprecipitation assay
ROS: reactive oxygen species
RT-(q)PCR: reverse transcription (quantitative) polymerase chain reaction
SC1/SC2: stem cell medium 1 or 2
SDS-PAGE: sodium dodecyl sulfate-polyacrylamide gel electrophoresis
SI: stain index
SNAI1/SNAI2: Snail Family Transcriptional Repressor 1/2
SNP: single nucleotide polymorphism
TCGA: The Cancer Genome Atlas
TC: tumor control (post-injection)
TTR: tumor take rate
tv: transcript variant
TWIST1: twist family BHLH transcription factor 1
VF2: normal foreskin-derived fibroblasts
VIM: vimentin
WB: Western blot(ting)
Wnt: wingless integrated (protein/pathway)
ZEB1/ZEB2: zinc-finger E-box-binding homeobox 1 or 2
