# Supplementary material for: Microenvironmentally-driven Plasticity of CD44 isoform expression determines Engraftment and Stem-like Phenotype in CRC cell lines
Source: Theranostics. 2020 Jun 18;10(17):7599–621. doi: 10.7150/thno.39893 (PMC7359088; doi:10.7150/thno.39893)
Supplement: Supplementary file 1 — Supplementary figures and tables. [file thnov10p7599s1.pdf]

Table S1

Antibodies (Ab) applied for flow cytometry (FC), fluorescence microcopy (FM) and Western blotting (WB)

aa - amino acids, n.d. - not defined, Gt - goat, Ms - mouse, Rb - rabbit, Rt - rat, Sw - swine, m - monoclonal, p - polyclonal, O/N - over night, RT - room temperature

§ the dilution is given if the concentration of the antibody stock solution is not specified by the manufacturer
* the signal was enhanced by a two-step Fluorescence Amplification by Sequential Employment of Reagents (FASER; Miltenyi Biotec) for FC (and FACS)
‡ for CD44 detection in SW620, αCD44 (2) was incubated with αMs IgG-HRP and subsequently probed with αRb IgG-HRP

Primary Ab against	Clone	Epitope	Host/ Clonality	Isotype	Conju-gation	Specificity	Distributor	Appli-cation	Working conc. (µg/mL)	Incubation conditions	Terminology in manuscript
CD44	MEM-85	n.d.	Ms/m	IgG2b	APC or PE	human	Immunotools	FC, FM	1:5§	45 min, 4 °C (FC)<br>2 h, RT (FM)	CD44-APC<br>CD44-PE
CD133	AC133	CD133/1	Ms/m	IgG1	PE	human	Miltenyi Biotec	FC	16.5	45 min, 4 °C	CD133-PE
CD326	HEA-125	n.d.	Ms/m	IgG1	FITC	human	Miltenyi Biotec	FC	1:20§	45 min, 4 °C	CD326-FITC
CD44v9	RV3	n.d.	Rt/m	IgG2a	-	human	Abnova	FC	10	1 h, 4 °C	CD44v9
CD44pan	EPR1013Y	aa153-171 of hCD44	Rb/m	IgG	-	human	Abcam	WB	1:1000§	O/N, 4°C	αCD44 (1)
CD44pan‡	156-3C11	constant part of hCD44	Ms/m	IgG2a	-	human	Cell Signaling	WB	1:1000§	O/N, 4 °C	αCD44 (2)
CD31	MEC 13.3	n.d.	Rt/m	IgG2a	-	mouse	*G. Breier, TU Dresden*	FM	1000	2 h, RT	CD31
β-actin	AC-15	aa1-15	Ms/m	IgG1	-	various	Abcam	WB	3100	O/N, 4 °C	β-Actin
α-tubulin	DM1A	aa 426-450	Ms/m	IgG1	-	various.	Millipore	WB	1000	O/N, 4 °C	α-Tub
MHCI+HLA B	EP2624	n.d.	Rb/m	IgG	-	human	Abcam	WB	2000	O/N, 4 °C	HLA-B
Pimonidazole	n.d.	n.d.	Rb/p	IgG	-	-	hpi	FM	200	2 h, RT	pimonidazole
**Isotype Ab**											
IgG1	IS5-21F5	n.d.	Ms/m	IgG1	FITC	n.d.	Miltenyi Biotec	FC	1:100§	45 min, 4 °C	Isotype
IgG1	IS5-21F5	n.d.	Ms/m	IgG1	PE	n.d.	Miltenyi Biotec	FC	22	45 min, 4 °C	Isotype
IgG2a	RTK2758	n.d.	Rt/m	IgG2a	-	n.d.	Abcam	FC	10	45 min, 4 °C	Isotype
IgG2b	PLRV219	n.d.	Ms/m	IgG2b	APC or PE	n.d.	Immunotools	FC<br>FM	1:100§ (FC)<br>1:5§ (FM)	45 min, 4 °C (FC)<br>2 h, RT (FM)	Isotype
**Secondary Ab against**											
Ms IgG	n.d.	n.d.	Gt/p	IgG	A488	mouse	Invitrogen	FC	10	30 min, 4 °C	
Ms IgG	n.d.	n.d.	Rb/p	IgG	HRP	mouse	Dako	WB	1300	1 h, RT	
Rb IgG	n.d.	n.d.	Sw/p	IgG	HRP	rabbit	Dako	WB	340	1 h, RT	
Rt IgG	n.d.	n.d.	Gt/p	IgG	A405	rat	Abcam	FC	1:2000	30 min, 4 °C	
Rt IgG	n.d.	n.d.	Gt/p	IgG	A488	rat	Life Technologies	FM	2000	2 h, RT	
Rt IgG	n.d.	n.d.	Gt/p	IgG	A594	rat	Life Technologies	FM	2000	2 h, RT	

Table S2

Primers used for the detection of *CD44*, its splice variants and the house keeping genes *ACTB (β-Actin)* and *B2M* (*β2-microglobulin*); primers were designed for human specificity

**Target gene**	**Primer sequence** 5'-----3'	**Number of cycles**	**Product size (bp)**
ACTB	Forward - CACCCTGAA GTACCCCATCG<br>Reverse - GCTGGGGTGTTGAAGGTCTC	20	199
B2M	Forward - AGGCTATCCAGCGTACTCCA<br>Reverse - TCAATGTCGGATGGATGAAA	20	112
CD133	Forward - GGATTATTCTATGCTGTGTCCTG<br>Reverse - TGCCACAAAACCATAGAAGAT	-	215
CTNNB1	Forward - ATTTGATGGAGTTGGACATGGC<br>Reverse - TGAGTGAAGGACTGAGAAAATCCC	-	211
ESRP1	Forward - ACAGAATGCGTTGAGGAAGC<br>Reverse - AGAGGGGCCGAGGAGAAT	-	120
ESRP2	Forward - AGGAGATGAGCCGAGTGCT<br>Reverse - GCTTGGAAGGTGGTGTAGGT	-	108
MMP2	Forward - GTGACGGAAAGATGTGGTGT<br>Reverse - CCAAATGAACCGGTCCTTGA	-	365
MMP9	Forward - GGCTTAGATCATTCCTCAGTG<br>Reverse - CTGCGGTGTGGTGGTGGTTG	-	365
SNAI1	Forward - GAAAGGCCTTCAACTGCAAA<br>Reverse - TGACATCTGAGTGGGTCTGG	-	249
SNAI2	Forward - TCGGACCCACACATTACCTT<br>Reverse - TGAGCCCTCAGATTTGACCT	-	159
TWIST1	Forward - CTCGGTCTGGAGGATGGAG<br>Reverse - CCACGCCCTGTTTCTTTGAA	-	228
VIM	Forward - CAGGCTCAGATTCAGGAACAG<br>Reverse - GGCGTCATTGTTCCGGTTGG	-	191
ZEB1	Forward - GGCATACACCTACTCAACTACGG<br>Reverse - TGGGCGGTGTAGAATCAGAGTC	-	155
ZEB2	Forward - AAGCCCCATCAACCCATACAAG<br>Reverse - AAATTCCTGAGGAAGGCCCA	-	124
CD44 total*	A, Forward - GTGATCAACAGTGGCAATGG<br>B, Reverse - CCACATTCTGCAGGTTCCTT	27	163
CD44 isoforms	C, Forward - GAAAGGAGCAGCACTTCAGG<br>D, Reverse - TGGAATTTGGGGTGTCCTTA	30	1392<br>1263<br>645<br>453<br>249<br>186
CD44 v9exon	C, Forward - GAAAGGAGCAGCACTTCAGG<br>E, Reverse - CAAGCCTTCATGTGATGTAGAG	30	1009<br>880<br>262

*Note: This primer set detects all CD44 transcript variants except for Tr. variant 8 (RefSeq: NM_001202557.1)

Table S3

Fractions of CD133⁺ and CD44⁺ cells in exponentially growing CRC cell lines with fluorescence signals above isotype controls determined in N≥3 independent experiments as representatively shown in Figures 1 and S1.

The list is arranged according to an increasing CD133⁺ cell fraction. The CD133 signal was selectively enhanced by a two-step FASER protocol (see Materials & Methods). CD24⁺ cell fractions were also analyzed and are given for completeness.

Cell line	Organ of origin	MSI/MSS status	CD133⁺ ± SD (%)*	CD44⁺ ± SD (%)*	CD133⁺/CD44⁺ ± SD (%)*	CD24⁺ ± SD (%)*	CD133⁺/ CD24⁺ ± SD (%)*
**RKO**	colon	MSI	0.0 ± 0.0	99.9 ± 0.2	0.0 ± 0.1	0.0 ± 0.0	0.0 ± 0.0
**SW480**	colon	MSS	0.2 ± 0.2 †	98.2 ± 2.7	0.6 ± 0.3	0.1 ± 0.2	0.2 ± 0.2
**SW837**	rectum	MSS	0.3 ± 0.2	86.4 ± 4.7	0.8 ± 0.9	0.2 ± 0.1	0.2 ± 0.1
**HCT-8**	colon	MSI	0.6 ± 0.5	88.9 ± 4.9	0.6 ± 0.4	0.8 ± 0.7	0.3 ± 0.2
**NCI-H716**	cecum	MSS	0.6 ± 0.3	77.2 ± 6.6	0.7 ± 0.3	25.2 ± 2.5	0.7 ± 0.3
**LS180**	colon	MSI	1.0 ± 0.3	99.7 ± 0.1	0.8 ± 0.2	10.8 ± 4.7	0.3 ± 0.1
**Colo-320 HSR**	colon	MSS	1.7 ± 1.1	94.0 ± 7.7	2.0 ± 1.2	0.9 ± 0.5	0.4 ± 0.5
**Colo-320 DM**	colon	MSS	3.1 ± 0.6	61.6 ± 9.4	1.5 ± 1.0	1.1 ± 0.9	0.0 ± 0.1
**SNU-C1**	colon	MSS	3.1 ± 1.5	98.2 ± 0.9	3.3 ± 0.9	42.3 ± 13.8	4.7 ± 0.0
**LS1034**	cecum	MSS	38.5 ± 10.9 †	0.2 ± 0.1	0.1 ± 0.2	87.6 ± 7.3	37.2 ± 11.6
**NCI-H630**	rectum	MSI	46.5 ± 4.5	37.8 ± 11.6	13.0 ± 6.0	77.8 ± 8.8	36.6 ± 5.9
**SW403**	colon	MSS	41.9 ± 4.7	97.0 ± 1.7	40.9 ± 4.5	0.3 ± 0.3	0.6 ± 0.1
**SW1417**	colon	MSS	69.7 ± 9.1 †	92.4 ± 3.6	52.7 ± 4.8	63.3 ± 7.4	33.6 ± 10.0
**SW620**	colon	MSS	73.5 ± 3.0 ‡	70.1 ± 1.5	51.2 ± 8.6	36.1 ± 6.6	26.9 ± 8.9
**HCT-116**	colon	MSI	78.0 ± 4.4 ‡	99.4 ± 1.6	80.5 ± 3.6	0.2 ± 0.1	0.1 ± 0.1
**Colo-201**	colon	MSS	89.2 ± 7.9	58.7 ± 14.0	58.8 ± 13.9	75.2 ± 16.6	72.5 ± 24.6
**CaCo2**	colon	MSS	94.9 ± 1.3	57.5 ± 3.5	59.9 ± 16.6	5.9 ± 3.7	4.6 ± 0.7
**HT29**	colon	MSS	97.3 ± 1.3 ‡	99.8 ± 0.2	96.5 ± 1.6	77.0 ± 7.5	82.1 ± 6.5
**LS513**	cecum	MSS	96.0 ± 0.7	94.0 ± 1.4	89.9 ± 1.0	8.7 ± 3.6	3.2 ± 2.3
**LS411N**	cecum	MSS	98.9 ± 0.2	99.0 ± 0.3	98.1 ± 0.8	83.7± 4.5	82.0 ± 7.2

* Note: Membrane-defect (PI-positive) cells were excluded in the analysis and polynomial gates were applied to best distinguish marker-positive from putatively negative cells. However, it is important to emphasize that all cell fractions are defined relative to cells stained with an isotype control antibody and neither necessarily nor always represent distinct subpopulations as often stated in the literature. For example, only 39% ± 11% of the LS1034 cells express a CD133⁺ fluorescence signal higher than the isotype control; howver, the fluorescence distribution of the entire population is shifted to the right (see Fig. S1) indicating that - based on flow cytometry best practice - there is only one population, and in principle all LS1034 cells might be slightly positive for CD133. This is invisible without (multiple) FASER amplification steps.

† The CD133 (but not CD44) data have been published earlier in *Peickert et al.* [33]

‡ CD133 subpopulations resemble previous measurements in the respective cell line using the advanced CD133 staining protocol [23,33]

Table S4

Significance levels (p values) for differences in the engraftment (tumor take rates, TTR) of cell lines (A) and cell line subpopulations defined by their CD44/CD133 surface expression profiles (B-D)

Statistical significance was evaluated by a bootstrapping approach as detailed in the Materials and Methods section; *control = run-through sorter original cells (processed according to the respective subpopulations)

(A) Comparison of cell line-specific TTR after injection of 10-10,000 *in vitro* grown cells (data documented in Figure 1B)

Cell line 1	*vs.*	Cell line 2	p
SW480		SW620	<0.01
SW480		LS1034	n.s.
SW620		LS1034	<0.001

(B) Comparison of TTR after injection of 10-100 *in vitro* grown SW620 cells sorted according to their CD133/CD44 surface expression pattern (data documented in Figure 2B)

Subpopulation 1	*vs.*	Subpopulation 2	p
$CD133^-/CD44^-$		$CD133^-/CD44^+$	n.s.
$CD133^-/CD44^-$		$CD133^+/CD44^-$	n.s.
$CD133^-/CD44^-$		$CD133^+/CD44^+$	n.s.
$CD133^-/CD44^+$		$CD133^+/CD44^+$	n.s.
$CD133^+/CD44^-$		$CD133^+/CD44^+$	n.s.
Control*		any CD133/CD44-defined subpopulation	n.s.

(C) Comparison of TTR after injection of 10-100 *in vitro* grown (CD44-negative) LS1034 cells sorted according to their CD133 surface expression (data documented in Figure 3B)

Subpopulation 1	*vs.*	Subpopulation 2	p
$CD133^-$		$CD133^+$	n.s.
Control*		$CD133^-$	0.057 (trend)
Control*		$CD133^+$	n.s.

(D) Comparison of TTR after injection of 500-10,000 LS1034 cells derived from xenografts and sorted according to their CD133/CD44 surface expression pattern (data documented in Figure 4B)

Subpopulation 1	*vs.*	Subpopulation 2	p
$CD133^-/CD44^-$		$CD133^+/CD44^-$	<0.02
$CD133^-/CD44^-$		$CD133^+/CD44^+$	<0.001
$CD133^+/CD44^-$		$CD133^+/CD44^+$	<0.01
Control*		$CD133^-/CD44^-$	<0.01
Control*		$CD133^+/CD44^-$	n.s.
Control*		$CD133^+/CD44^+$	<0.01

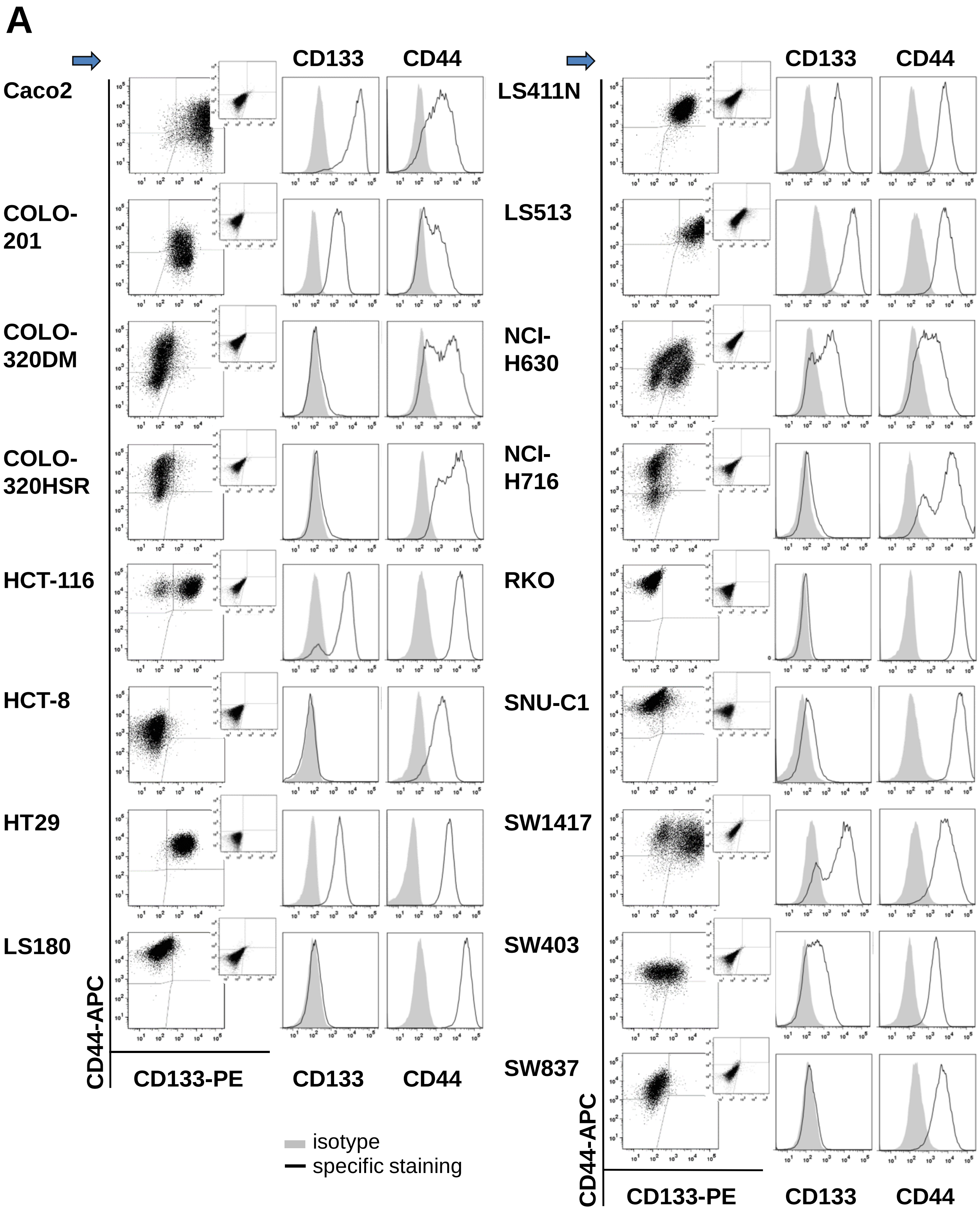

Figure S1 A

Representative flow cytometric dot blot diagrams and histograms showing the CD133 and CD44 surface pattern in various exponentially growing CRC cell lines kept under identical 2-D *in vitro* conditions. Cell lines are listed in alphabetical order; immunofluorescence stainings were performed with the antibodies and conditions given in Table S1. Notably, the CD133 (AC133) fluorescence signal was enhanced by a two step FASER series as detailed earlier [23,33].

B

$$\text{Stain index} = \frac{\text{median FL}_{\text{stained sample}} - \text{median Fl}_{\text{isotype}}}{\text{2xSD of Fl}_{\text{isotype}}}$$

FL - fluorescence signal
SD - standard deviation

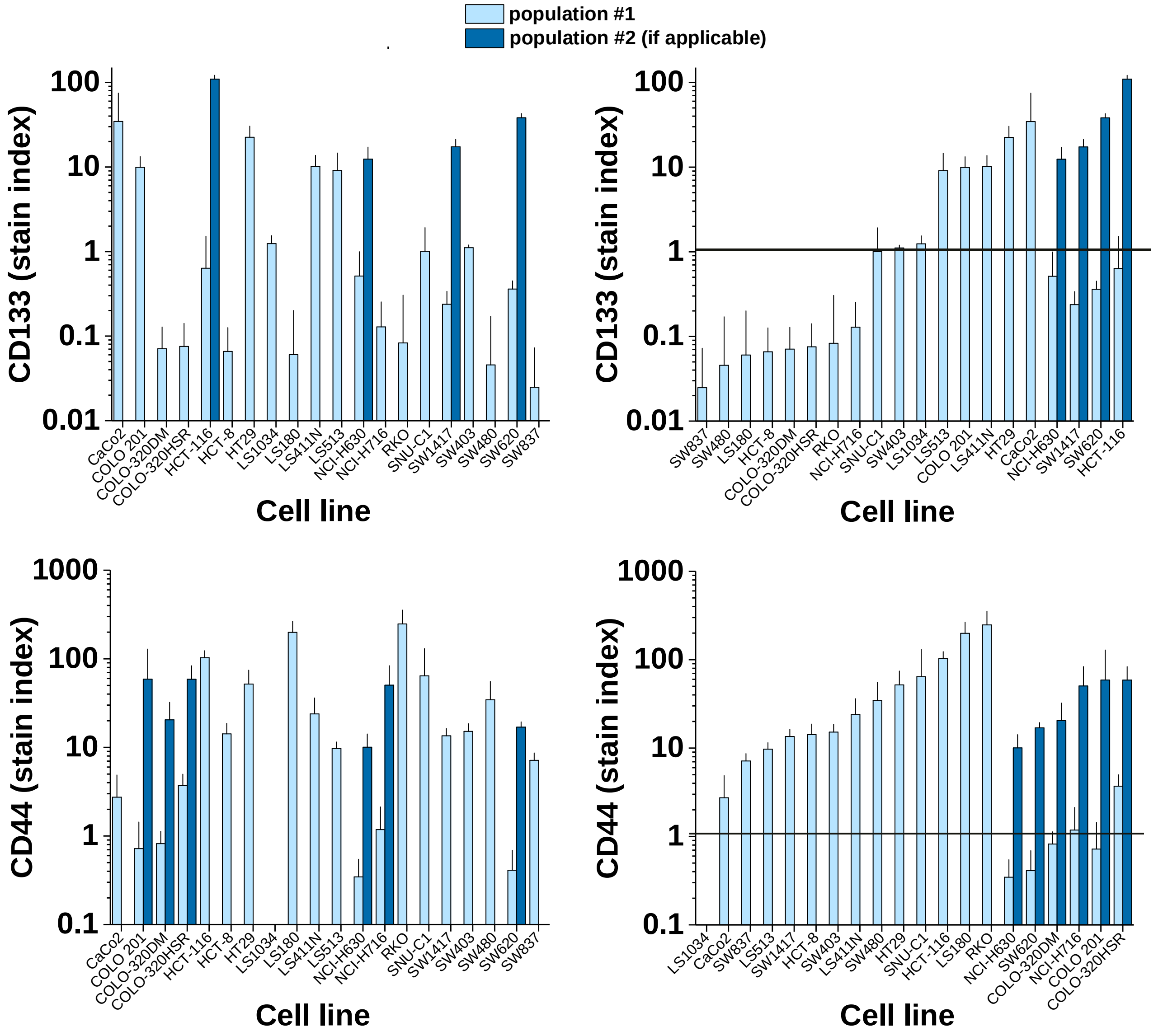

Figure S1 B

Stain index (SI) for CD133 and CD44 fluorescence signals in cell line subpopulations that could be clearly distinguished in the dot blot diagrams as representatively shown in (A). The graphs document average values +SD from N≥3 independent experiments. On the left, results are illustrated for cell lines alphabetically ordered according to Figure S1A; the right graphs display the data ordered by (i) an increasing SI which quantitatively reflects biomarker surface expression and (ii) the presence of one or two distinct populations.

Note: Populations can be categorized as follows: SI<1 = equivalent to the marker-negative isotype controls, SI>1 to 2 = marginal; SI>2 to 5 = low, SI>5 to 20 = intermediate, SI>20 to100 = high, and SI>100 = exceptionally high marker expression

A

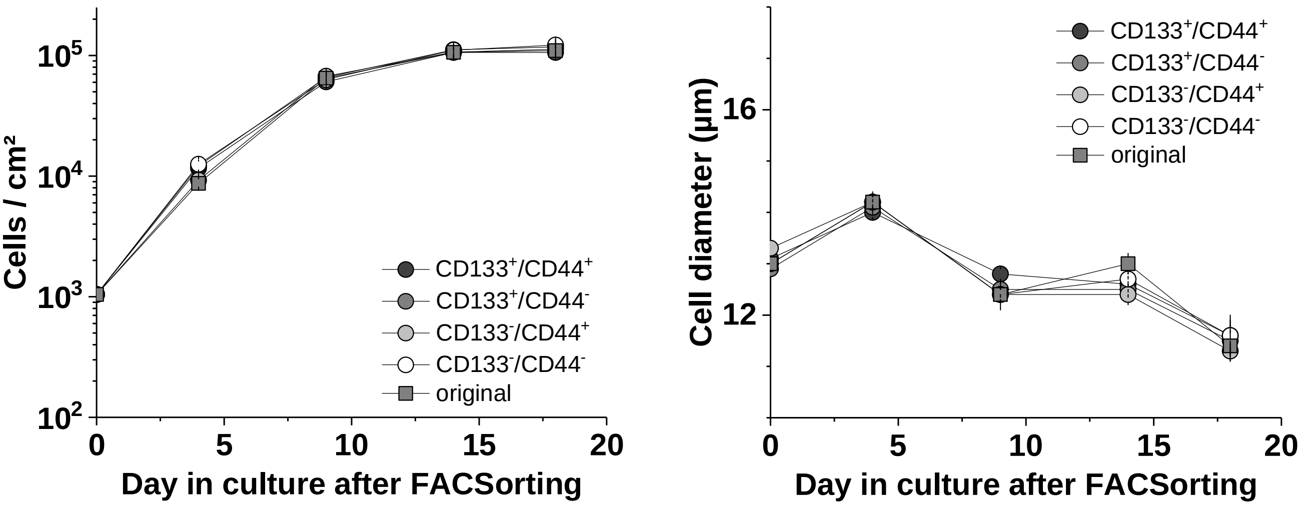

B

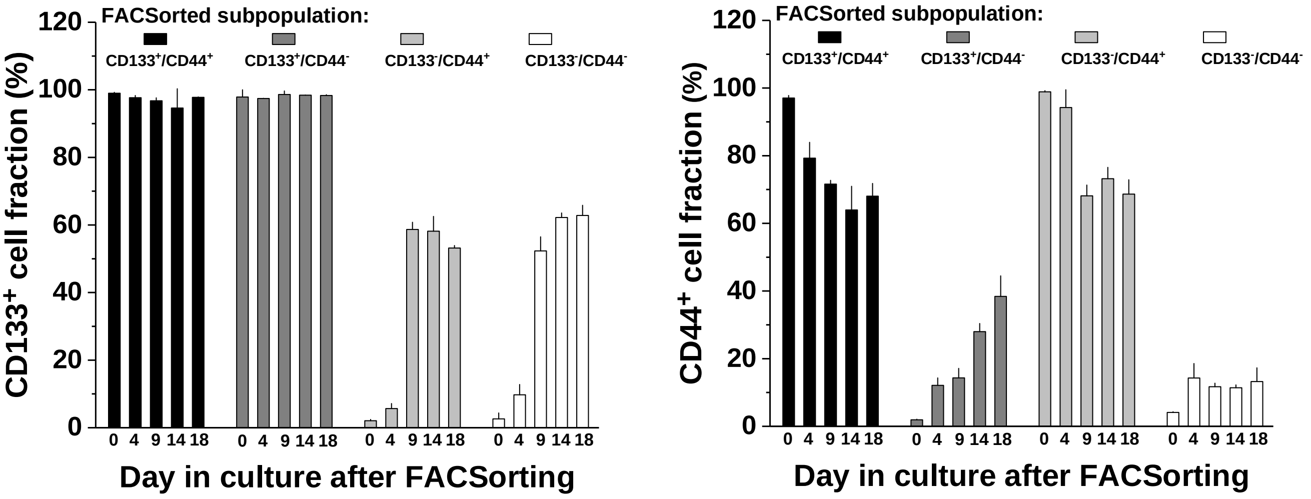

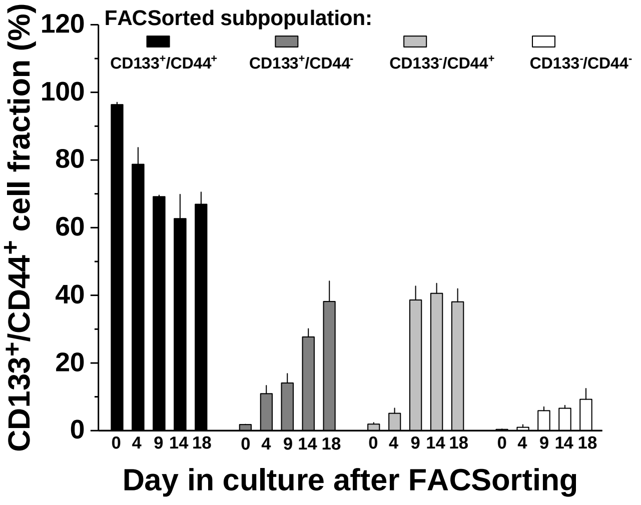

Figure S2 A/B

SW620 cell populations with distinct CD133/CD44 surface pattern *in vitro* do not differ in growth kinetics or cell morphology but differentially re-adapt their surface expression profile in culture.

(A) Growth behavior and modifications in mean cell diameter of SW620 subpopulations cultured up to 18 days after FACSorting; one experiment with intraexperimental variation is documented (N=1, n=3).

(**B)** Fractions of cells with $CD133^+$, $CD44^+$ and $CD133^+/CD44^+$ surface expression in cultures grown from the respective FACSorted SW620 subpopulations; average values ± SD from three independent experiments are shown (N=3; n=1-2).

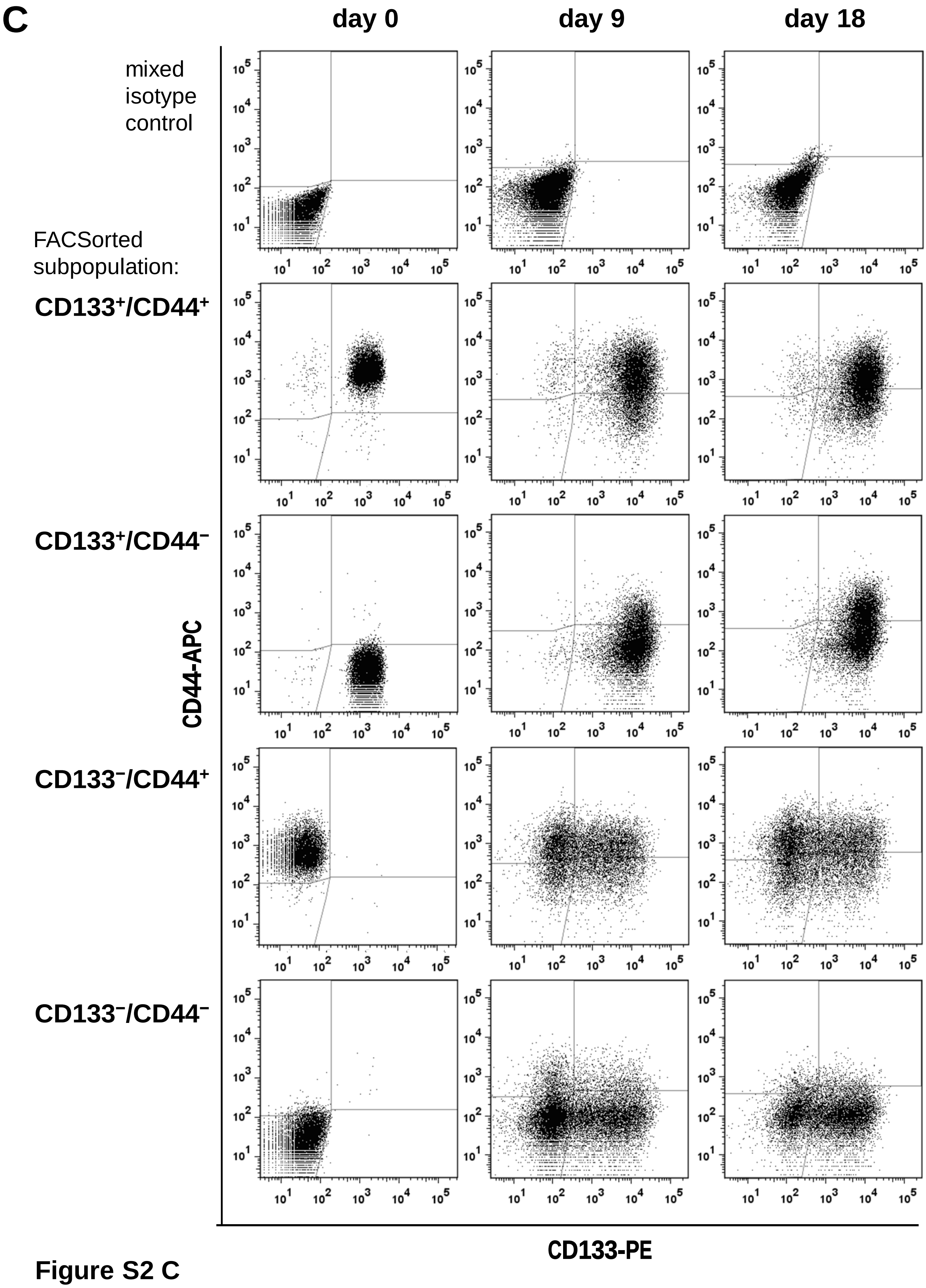

Figure S2 C

Representative flow cytometric CD133/CD44 dot blot diagrams from one experimental series included in the analyses shown in Figure S2B.

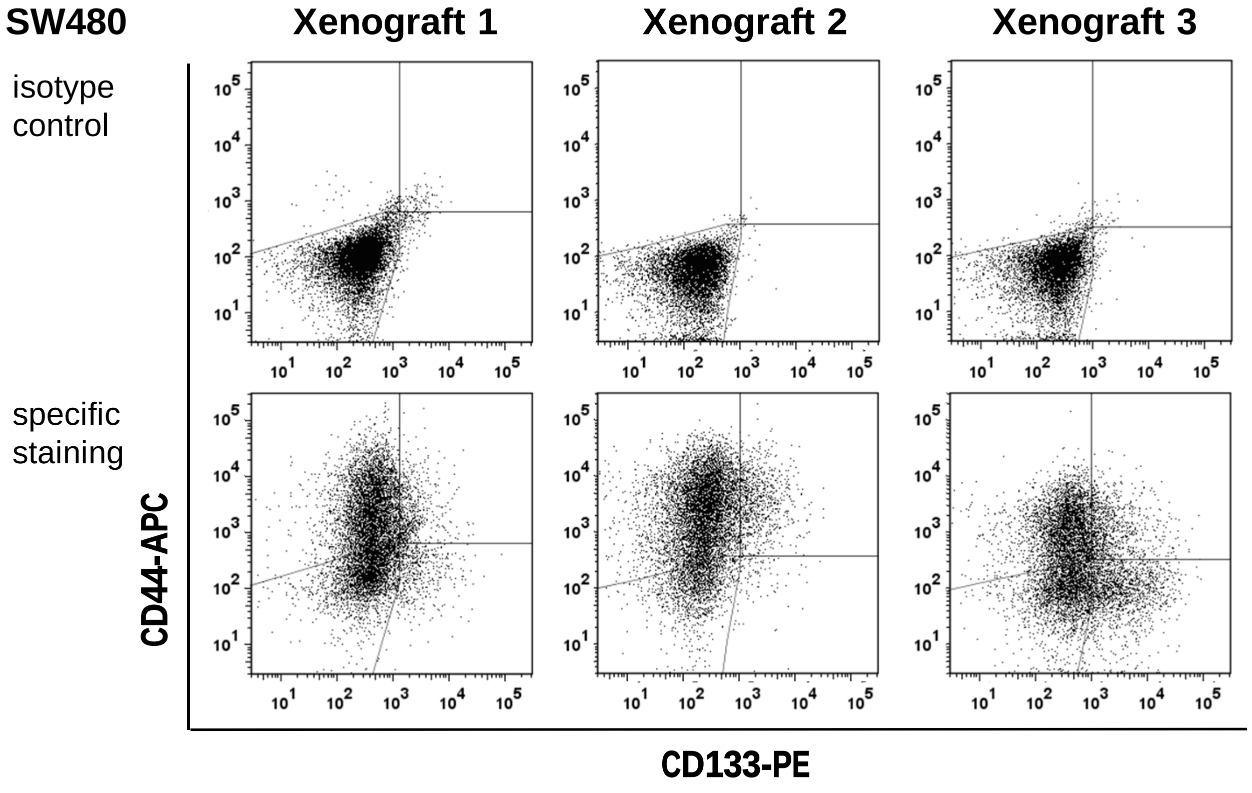

Figure S3

Flow cytometric dot blot diagrams showing the CD133/CD44 surface pattern in SW480 cell suspensions prepared from three individual xenografts. The samples were pre-gated to exclude non-human and membrane-defect (PI-positive) cells. Aliquots exposed to isotype control antibodies required for analyses of cell fractions are documented in the upper row. For comparison with (i) CD133/CD44 expression in SW480 monolayer culture (cf. Figure 1) and (ii) CD133/CD44 profile in SW620 xenograft cells (cf. Figure 2). Notably, in all SW480 xenograft preparations a minor proportion (0.1-1%) of cells stained with the isotype antibody shows FC signals beyond the major isotype gate limiting the sensitivity of quantitation.

A Median section of 1st generation LS1034 xenograft (#2)

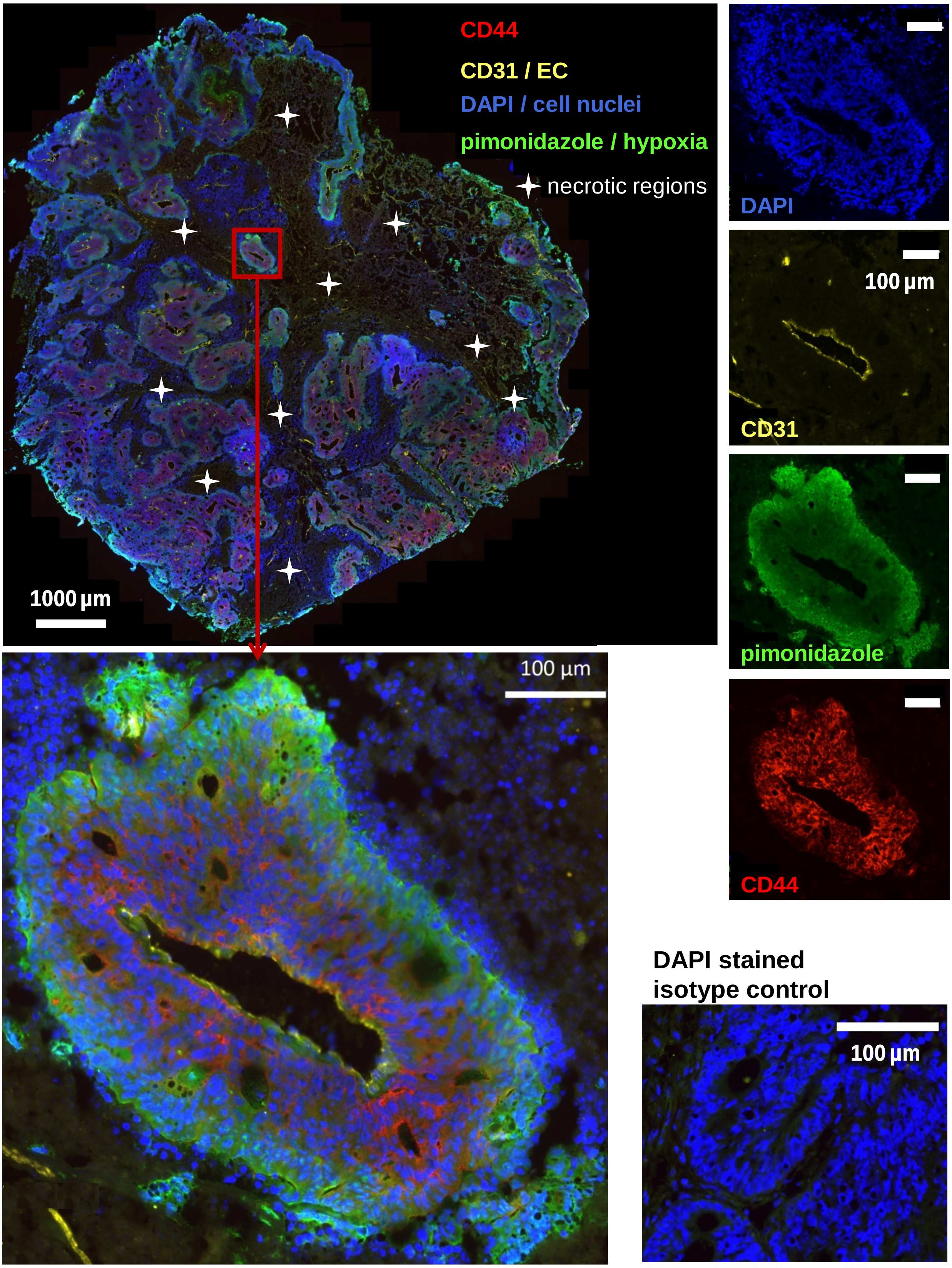

Figure S4 A/B

Median frozen sections (10 µm) of two additional LS1034 xenografts co-stained for CD44, CD31 (endothelial cells), pimonidazole (hypoxia) and DAPI (nuclei) and imaged with a magnification of 200x support the finding highlighted in Figure 5 in spite of histomorphological heterogeneity and staining variations. Whole tumor sections (stitched from >1,000 single images - top) as well as selected regions at higher magnification (bottom) are displayed as four-channel overlay; bars represent 1,000 µm in whole tumor sections and 100 µm in all other images.

B Median section of 1st generation LS1034 xenograft (#3)

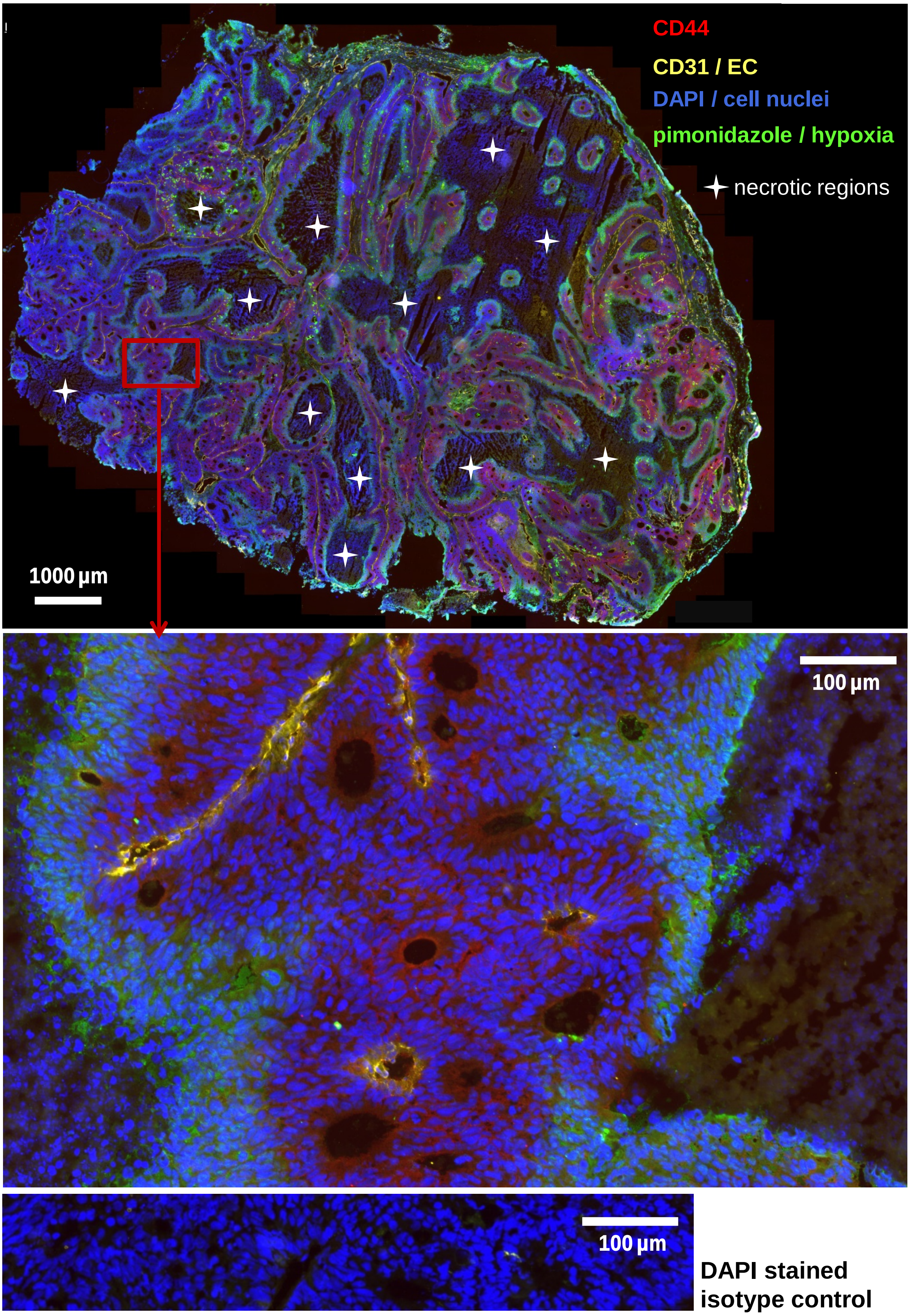

DAPI stained isotype control

Figure S4 A/B (continued)

Representative parts of sections stained with isotype antibodies and DAPI are shown as controls (multi-channel).
In **(A)**, single fluorescence channel images of the magnified region are also depicted (right).

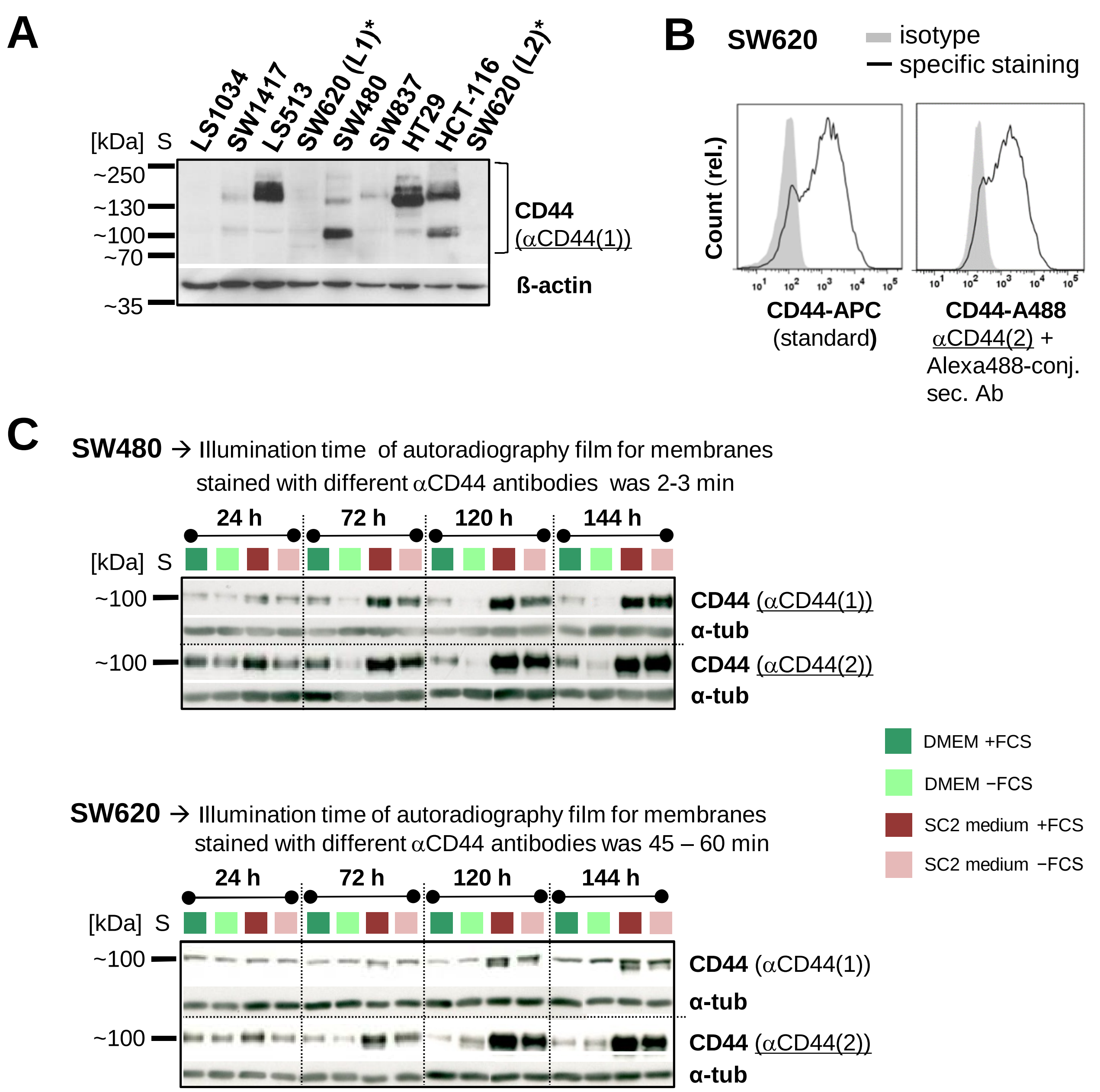

Figure S5

Heterologeous CD44 protein expression in CRC cell lines as detected by Western blotting (WB); 40 µg (A) and 25 µg (C) of total protein was loaded per lane. No WB bands were detected with any of the antibodies and illumination times for LS1034 cells grown under diverse conditions (negative blots not shown). Flow cytometric analyses (FC) reveal CD44 surface presentation on SW620 cells.

(A) Representative WB of CD44 pattern in whole cell protein extracts of various CRC cell lines as detected with the pan-αCD44(1) antibody (Ab); *two independly prepared protein lysates of SW620 monolayer cells (L1, L2) were loaded on this specific SDS-PAGE.

(B) CD44 surface presentation in SW620 monolayer cells as detected by FC (cf. Figures 1, 2 and S1) is confirmed with a second pan-αCD44 (αCD44(2)).

(C) Representative WBs showing the main CD44 protein band at ~100-110 kDa via two different pan-αCD44 Abs (αCD44(1) and αCD44(2)) in SW480 and SW620 cells grown for 24-144 h in DMEM or stem cell medium (SC2) with and without serum.

A

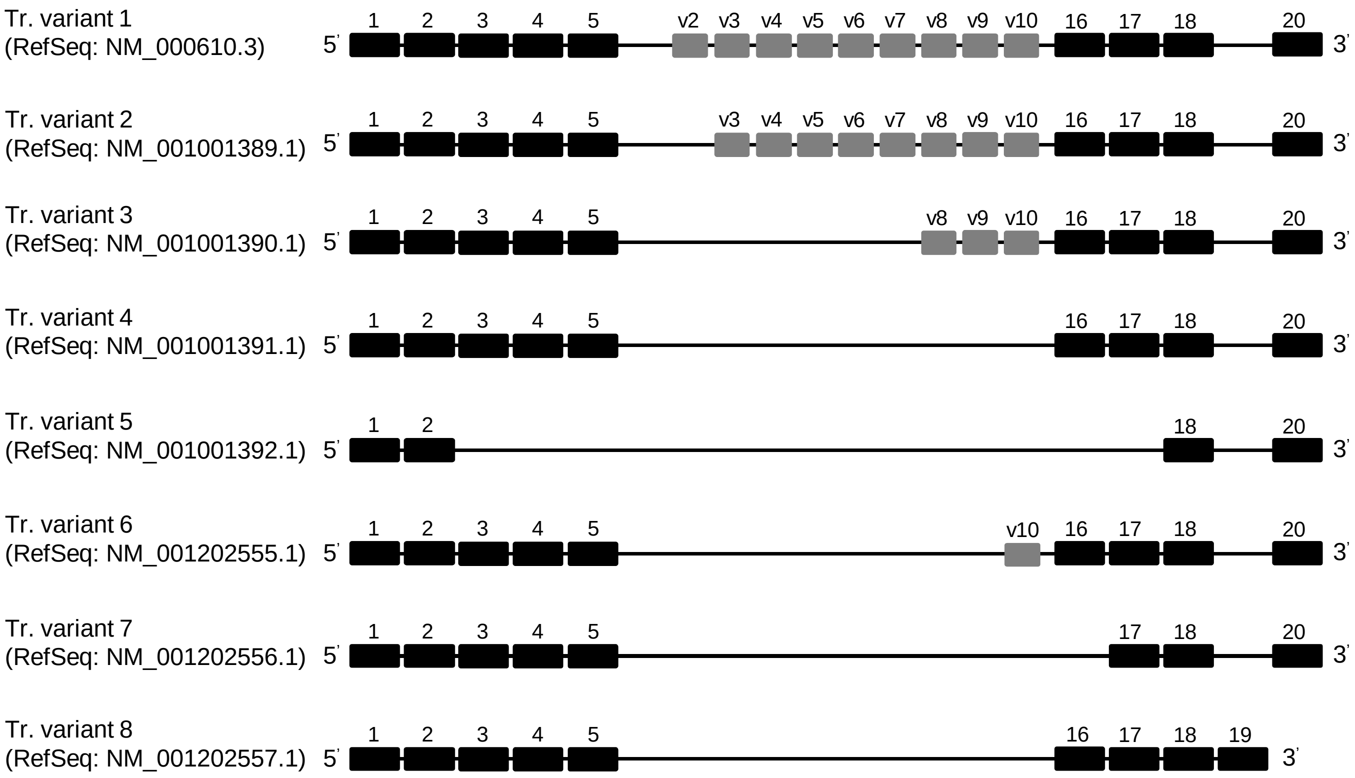

Figure S6 A-C

(A) Overview of human *CD44* transcript variants 1-8 with their constant and variable exons. Notably, our PCR design and primers (cf. Figure 5C-F) allowed to detect all variants except for *Tr. variant 8* while the sequencing could not discriminate *Tr. variants 4* and *8*. The product of *Tr. variant 4* is CD44s which is likely to be expressed in CRC cells; *Tr. variant 8* supposedly translates into a more rare short-tail or tail-less CD44 isoform (CD44st) as exon 18 contains a stop codon that originates a truncated cytoplasmic tail, consequently leading to the loss of intracellular protein domains and signaling motifs necessary for the interaction with cytoskeletal components [46]; function and relevance of this isoform are unclear and thus not further discussed

(B/C) *CD44* product sequence chromatograms and sequences, respectively, for PCR products obtained with primer pairs C/D (645 bp and 249 bp) and C/E (262 bp) according to Figure 5E/F (see next 3 pages)

★monoallelic mutation in exon v8 (c.689T>C [p.Ile230Thr] identified in the 645 bp (primer pair C/D) and the 262 bp (primer pair C/E) sequence products according to *CD44* transcript mRNA isoform 3 (RefSeq: NM_001001390.1)

B

CD44 645 bp product sequence chromatogram: reading with forward primer C

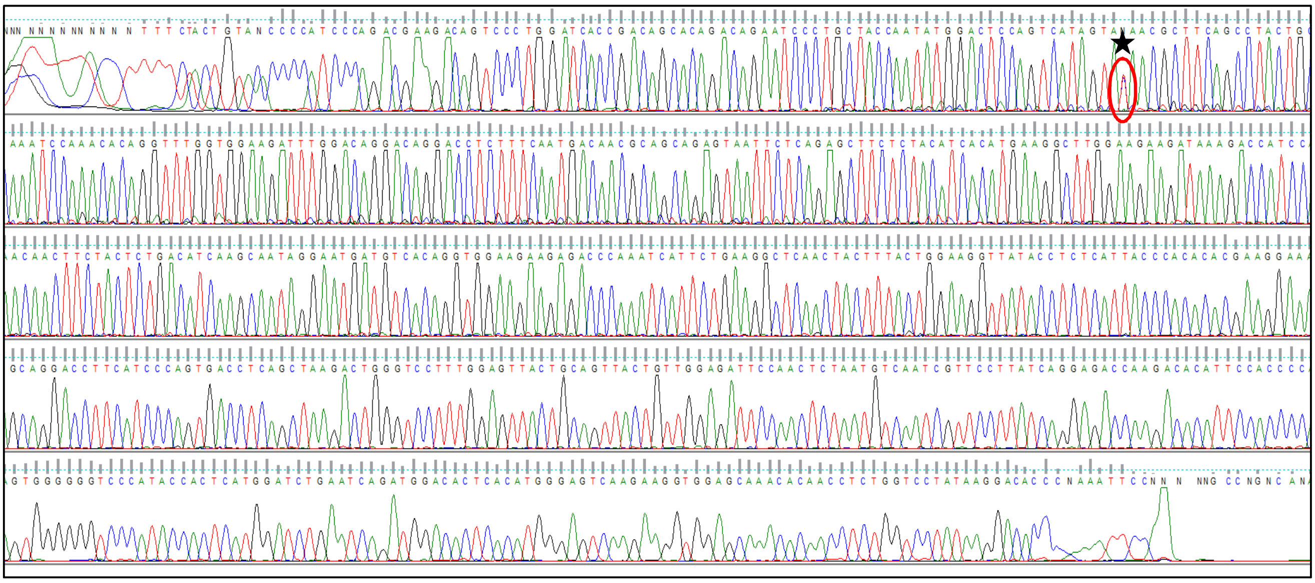

CD44 645 bp product sequence chromatogram : reading with reverse primer D

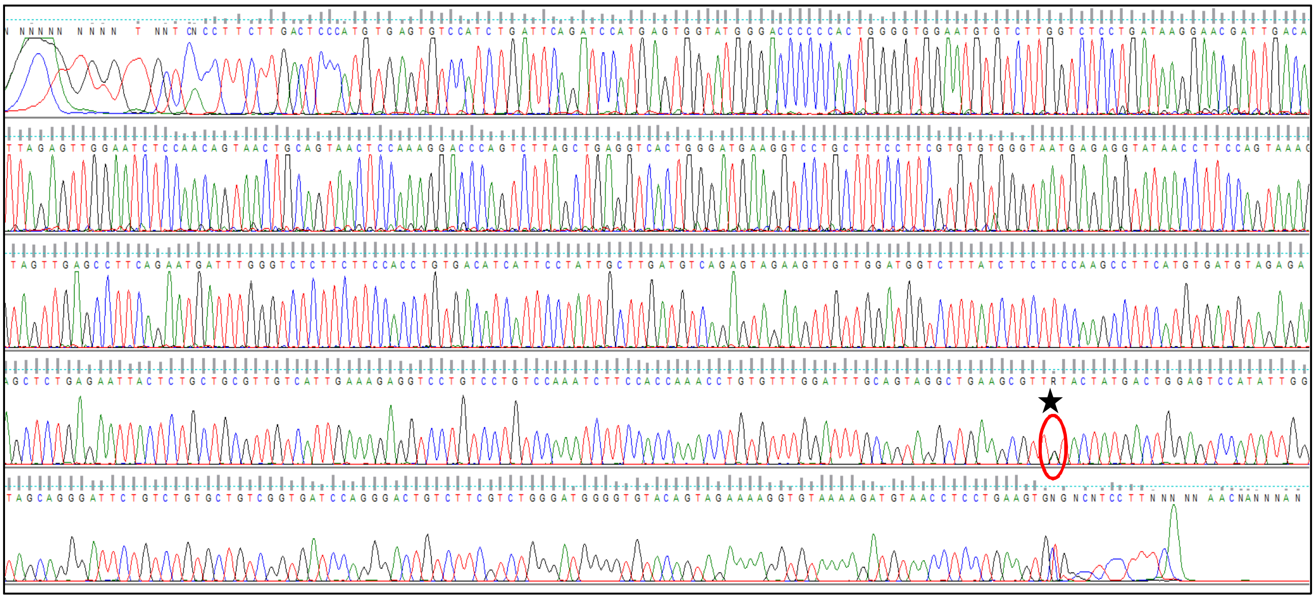

Sequence of *CD44* 645 bp PCR product from sequencing experiments
(RefSeq: NM_001001390.1; BLAST Alignment 987..1631 bp)

GAAAGGAGCAGCACTTCAGGAGGTTACATCTTTTACACCTTTTCTACTGTACACCCCA
TCCCAGACGAAGACAGTCCCTGGATCACCGACAGCACAGACAGAATCCCTGCTACC
AATATGGACTCCAGTCATAGTA**N**AACGCTTCAGCCTACTGCAAATCCAAACACAGGTT
TGGTGGAAGATTTGGACAGGACAGGACCTCTTTCAATGACAACGCAGCAGAGTAATT
CTCAGAGCTTCTCTACATCACATGAAGGCTTGGAAGAAGATAAAGACCATCCAACAAC
TTCTACTCTGACATCAAGCAATAGGAATGATGTCACAGGTGGAAGAAGAGACCCAAAT
CATTCTGAAGGCTCAACTACTTTACTGGAAGGTTATACCTCTCATTACCCACACACGA
AGGAAAGCAGGACCTTCATCCCAGTGACCTCAGCTAAGACTGGGTCCTTTGGAGTTA
CTGCAGTTACTGTTGGAGATTCCAACTCTAATGTCAATCGTTCCTTATCAGGAGACCA
AGACACATTCCACCCCAGTGGGGGGTCCCATACCACTCATGGATCTGAATCAGATGG
ACACTCACATGGGAGTCAAGAAGGTGGAGCAAACACAACCTCTGGTCCTATAAGGAC
ACCCCAAATTCCA

B **continued**

CD44 249 bp product sequence chromatogram : reading with forward primer C

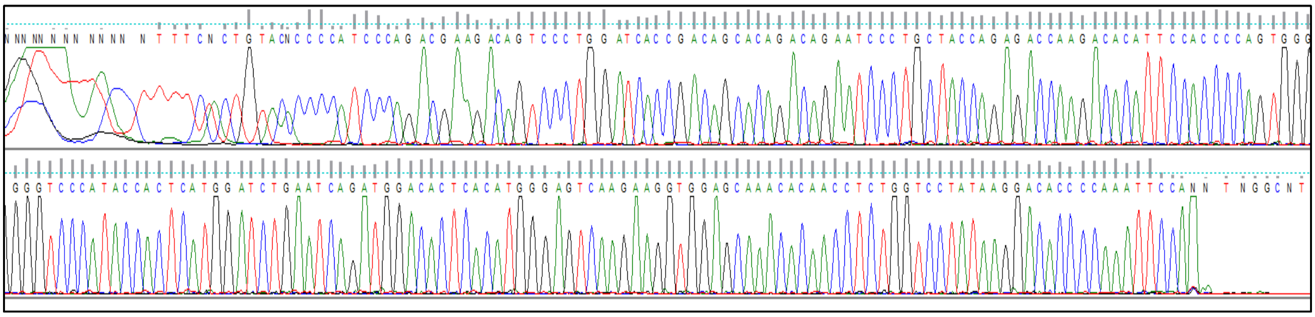

CD44 249 bp product sequence chromatogram : reading with reverse primer D

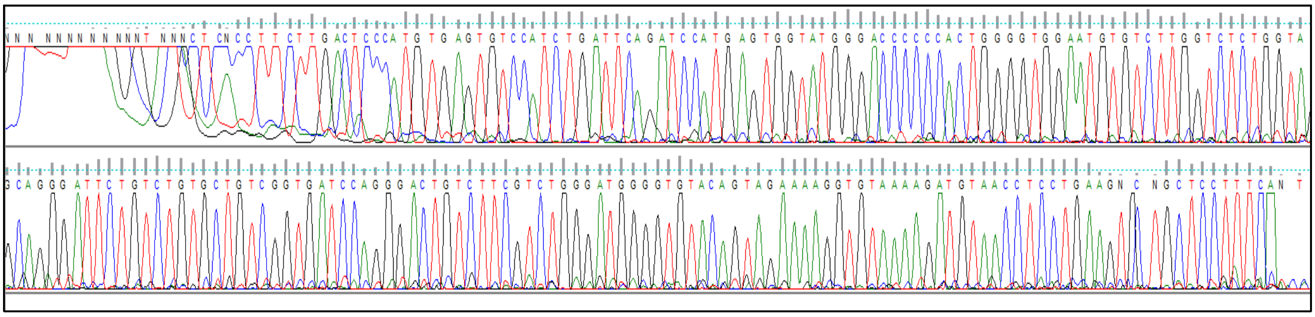

Sequence of *CD44* 249 bp PCR product from sequencing experiments
(RefSeq: NM_001001391.1 and NM_001202557.1; BLAST Alignment 987..1235 bp)

GAAAGGAGCAGCACTTCAGGAGGTTACATCTTTTACACCTTTTCTACTGTACACCCCA
TCCCAGACGAAGACAGTCCCTGGATCACCGACAGCACAGACAGAATCCCTGCTACC
AGAGACCAAGACACATTCCACCCCAGTGGGGGGTCCCATACCACTCATGGATCTGAA
TCAGATGGACACTCACATGGGAGTCAAGAAGGTGGAGCAAACACAACCTCTGGTCC
TATAAGGACACCCCAAATTCCA

C

CD44 262 bp product sequence chromatogram : reading with forward primer C

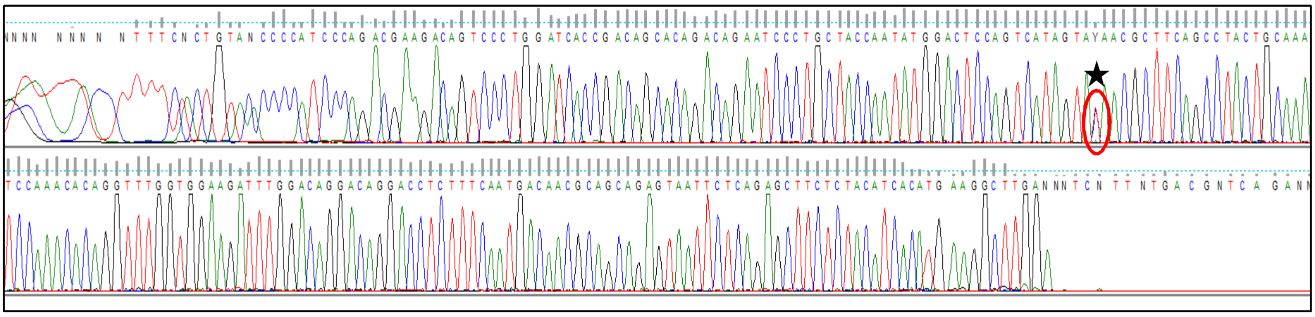

CD44 262 bp product sequence chromatogram : reading with reverse primer E

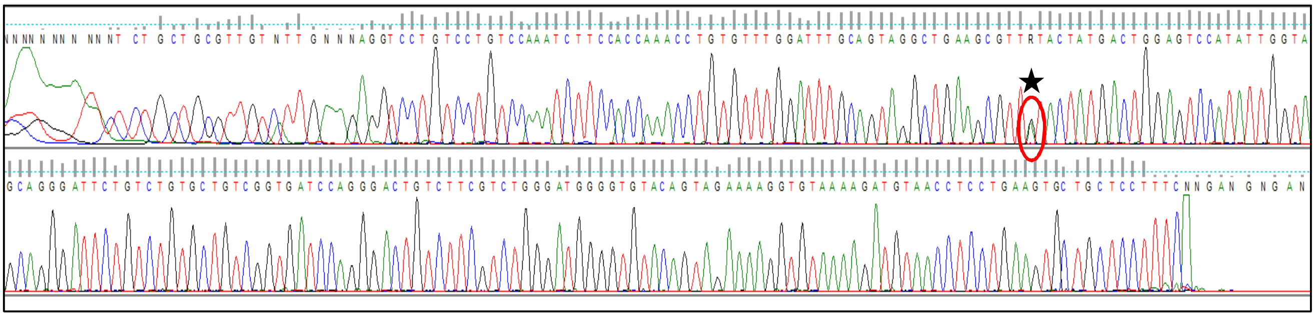

Sequence of *CD44* 262 bp PCR product from sequencing experiments
(RefSeq: NM_001001390.1; BLAST Alignment 987..1248 bp)

GAAAGGAGCAGCACTTCAGGAGGTTACATCTTTTACACCTTTTCTACTGTACACCCCA
TCCCAGACGAAGACAGTCCCTGGATCACCGACAGCACAGACAGAATCCCTGCTACC
AATATGGACTCCAGTCATAGTA**Y**AACGCTTCAGCCTACTGCAAATCCAAACACAGGTT
TGGTGGAAGATTTGGACAGGACAGGACCTCTTTCAATGACAACGCAGCAGAGTAATT
CTCAGAGCTTCTCTACATCACATGAAGGCTTGG

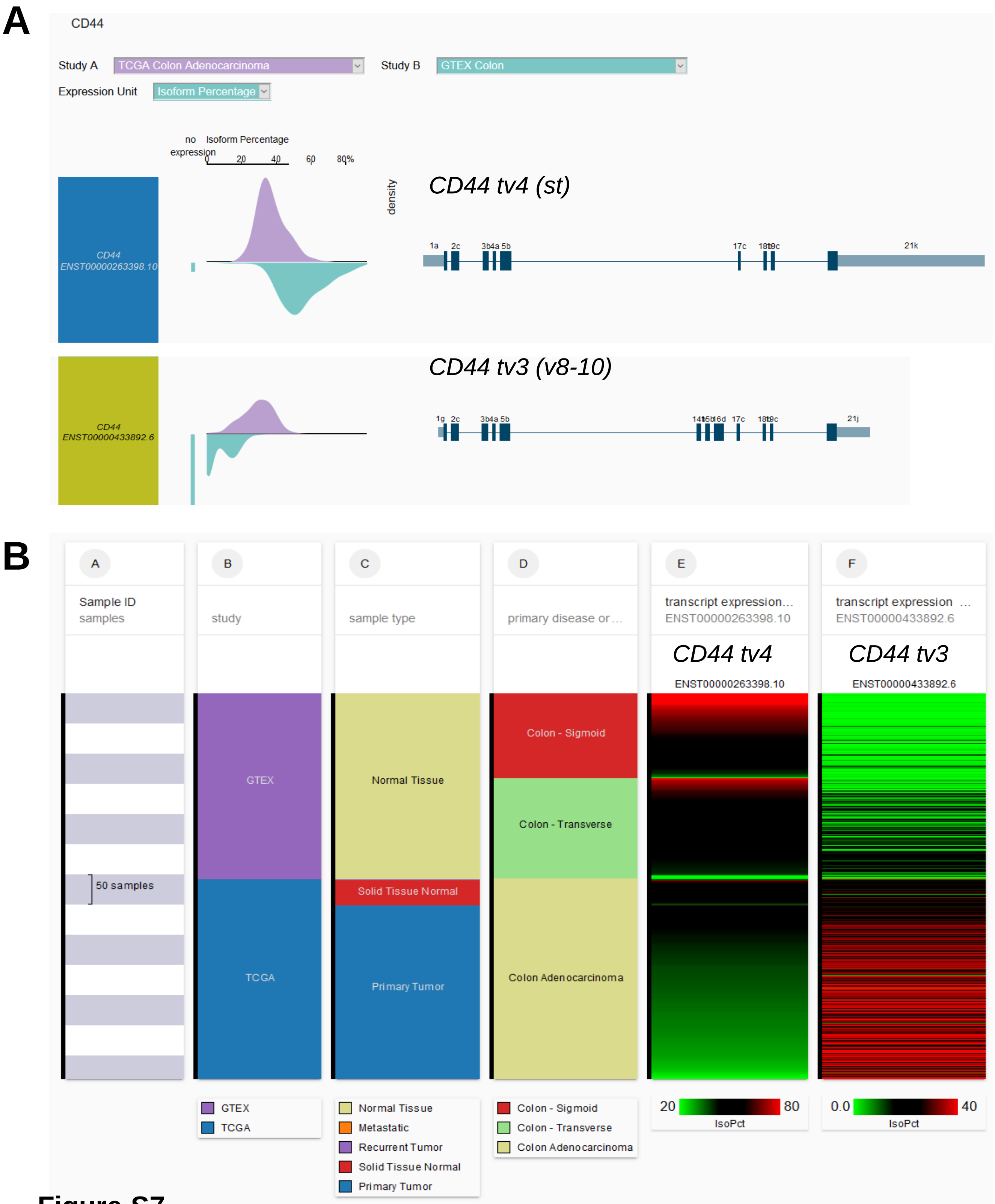

Figure S7

Schematic representation of inverse transcript expression of *CD44 tv4* (st) and *CD44 tv3* (v8-10) in primary normal colon epithelium and colon adenocarcinoma tissue based on the TCGA (n=308) and GTEX (n=331) databases.

(A) Visualization of the *CD44 tv3* and *CD44 tv4* transcript structures and density plots showing the expression range in the tissues of interest.

(B) Heatmap of *CD44 tv3* and *CD44 tv4* transcript-specific expression (% of isoform) in colon adenocarcinoma and primary normal colon epithelium
